# Sequence and Expression Analysis of Interferon Regulatory Factor 10 (IRF10) in Three Diverse Teleost Fish Reveals Its Role in Antiviral Defense

**DOI:** 10.1371/journal.pone.0147181

**Published:** 2016-01-19

**Authors:** Qiaoqing Xu, Yousheng Jiang, Eakapol Wangkahart, Jun Zou, Mingxian Chang, Daiqin Yang, Chris J. Secombes, Pin Nie, Tiehui Wang

**Affiliations:** 1 School of Animal Science, Yangtze University, Jingzhou, Hubei Province 434025, P. R. China; 2 Scottish Fish Immunology Research Centre, Institute of Biological and Environmental Sciences, University of Aberdeen, Aberdeen AB24 2TZ, United Kingdom; 3 State Key Laboratory of Freshwater Ecology and Biotechnology, Institute of Hydrobiology, Chinese Academy of Sciences, Wuhan, Hubei Province 430072, P. R. China; 4 College of Fishery and Life Science, Shanghai Ocean University, Shanghai 201306, China; INRA, FRANCE

## Abstract

**Background:**

Interferon regulatory factor (IRF) 10 was first found in birds and is present in the genome of other tetrapods (but not humans and mice), as well as in teleost fish. The functional role of IRF10 in vertebrate immunity is relatively unknown compared to IRF1-9. The target of this research was to clone and characterize the IRF10 genes in three economically important fish species that will facilitate future evaluation of this molecule in fish innate and adaptive immunity.

**Molecular Characterization of IRF10 in Three Fish Species:**

In the present study, a single IRF10 gene was cloned in grass carp *Ctenopharyngodon idella* and Asian swamp eel *Monopterus albus*, and two, named IRF10a and IRF10b, in rainbow trout *Oncorhynchus mykiss*. The fish IRF10 molecules share highest identities to other vertebrate IRF10s, and have a well conserved DNA binding domain, IRF-associated domain, and an 8 exon/7 intron structure with conserved intron phase. The presence of an upstream ATG or open reading frame (ORF) in the 5’-untranslated region of different fish IRF10 cDNA sequences suggests potential regulation at the translational level, and this has been verified by *in vitro* transcription/translation experiments of the trout IRF10a cDNA, but would still need to be validated in fish cells.

**Expression Analysis of IRF10 *In Vivo* and *In Vitro*:**

Both trout IRF10 paralogues are highly expressed in thymus, blood and spleen but are relatively low in head kidney and caudal kidney. Trout IRF10b expression is significantly higher than IRF10a in integumentary tissues i.e. gills, scales, skin, intestine, adipose fin and tail fins, suggesting that IRF10b may be more important in mucosal immunity. The expression of both trout IRF10 paralogues is up-regulated by recombinant IFN-γ. The expression of the IRF10 genes is highly induced by Poly I:C *in vitro* and *in vivo*, and by viral infection, but is less responsive to peptidoglycan and bacterial infection, suggesting an important role of fish IRF10 in antiviral defense.

## Introduction

Interferon (IFN) regulatory factors (IRFs) are ancient molecules that have been conserved throughout the 600 million years of metazoan evolution [1]. They were first and best characterized as transcriptional regulators of type I IFNs and IFN-inducible genes within the vertebrates [2, 3]. It is now recognized that IRFs play diverse roles in innate immune responses elicited by pattern recognition receptors (PRRs), in development of various immune cells, in the control of cell growth, cell survival and oncogenesis, and even in sex determination [2, 4, 5]. So far eleven vertebrate IRF family members (IRF1–11) have been described, with IRF11 being teleost fish-specific [6, 7].

All IRF family members possess an N-terminal DNA binding domain (DBD) and a C-terminal IRF-association domain (IAD). The DBD is characterized by a series of five relatively well-conserved tryptophan (W)-rich repeats and forms a helix-turn-helix structure. It recognizes a DNA sequence known as the IFN-stimulated response element (ISRE) which is characterized by the consensus sequence, 5'-AANNGAAA-3' [8]. The C-terminal IAD mediates the interactions of a specific IRF with other family members, other transcription factors, or cofactors, so as to confer specific activities upon each IRF. The activation of IRFs is triggered by phosphorylation in the C-terminal region which induces conformation changes permitting extensive contacts to a second subunit that is transported into the nucleus [9]. The interaction of IRFs with other transcription factors can further determine whether the resulting complex functions as a transcriptional activator or repressor, and defines the nucleotide sequences adjacent to the core IRF binding motif to which the transcriptional complex binds [2, 8].

Being present in humans and mice, IRF1-9 have been well studied and have diverse roles. For example, mammalian IRF1, IRF3, IRF4, IRF5, IRF7, and IRF8 have a crucial involvement in innate immune responses elicited by PRRs, and IRF1, IRF2, IRF4, and IRF8 play a role in the development of various immune cells [2, 8]. In contrast, the function of IRF10, that is present in the genomes of other mammals but is not found in humans and mice, is unknown in mammals. The first functional analysis of IRF10 was in birds, where chicken IRF10 up-regulates the expression of two IFN-γ induced target genes (major histocompatibility complex (MHC) class I and guanylate-binding protein) and interferes with the induction of the type I IFN induced gene 2’, 5’-oligo(A) synthetase [10]. The expression of the chicken IRF10 gene can be induced by both type I IFN (IFN1) and IFN-γ, as well as by infectious bursal disease virus [10, 11]. The IRF10 gene has also been identified in several model fish species, such as zebrafish *Danio rerio* [6], fugu *Takifugu rubripes*, medaka *Oryzias latipes*, stickleback *Gasterosteus aculeatus* [7] and Japanese flounder *Paralichthys olivaceus* [12]. Functional characterization of fish IRF10 has also been started recently. Polyinosinic:polycytidylic acid (Poly I:C) can induce IRF10 expression in Japanese flounder and zebrafish [12, 13]. Bacterial and viral infections have also been shown to induce Japanese flounder IRF10 expression in kidney. Over expression of zebrafish IRF10 inhibits the activation of some type I IFNs promoters and decreases the transcription level of some IFN-stimulated genes, suggesting that fish IRF10 may have a crucial role in immune defense [13]. However, the characterization of IRF10 function in more economically important aquacultured fish species is still absent.

In this report, we first cloned and characterized four IRF10 genes in three economically important fish species, one each in grass carp *Ctenopharyngodon idella* and Asian swamp eel *Monopterus albus* and two in the rainbow trout *Oncorhynchus mykiss*. We then examined their expression *in vivo* in healthy fish, and *in vitro* in rainbow trout primary macrophages stimulated with pathogen-associated molecular patterns (PAMPs) and recombinant cytokines. Poly I:C and IFN-γ were found to be strong inducers of trout IRF10 paralogues in primary macrophages. We further confirmed *in vivo* that Poly I:C can induce IRF10 expression in swamp eel, and viral infection can induce IRF10 expression in both grass carp and rainbow trout. Such results suggest a role of teleost IRF10 in antiviral defense.

## Material and Methods

### 2.1. Fish

Rainbow trout (~100 g), grass carp (~250 g), and eel (~70 g), were separately maintained in aerated fibreglass tanks supplied with a continuous flow of recirculating freshwater. Fish were fed twice daily on commercial pellets, and were given at least 2 weeks of acclimatization prior to treatment. All the experiments described comply with the Guidelines of the European Union Council (2010/63/EU) for the use of laboratory animals. The protocol was approved by the ethics committee at the University of Aberdeen and the work was carried out under project license PPL 60/4013.

### 2.2 Cloning of IRF10 in three aquacultured fish species

#### 2.2.1 Cloning of IRF10 in the Asian swamp eel and grass carp

No transcriptomic or genomic resources are available for swamp eel and grass carp. Thus homology cloning was employed for the cloning of IRF10 genes in these two species. Degenerate primers (Table 1) were designed against conserved IRF10 sequences from zebrafish, Japanese flounder, and chicken (GenBank accession nos. BC153602, AB359170 and AF380350, respectively), and used to obtain partial IRF10 cDNA sequences from each species. The cDNA samples used for cloning were prepared from isolated head kidney (HK) cells stimulated with 25 μg/ml of Poly I:C (Sigma) for 24 h. To obtain the full-length cDNA sequence, 3′-RACE and 5′-RACE were performed using gene-specific primers (Table 1) and SMART cDNA as described previously [14,15].

**Table 1:** Primers used for cDNA cloning and real-time PCR analysis of gene expression.

Gene	Primer	Sequences (5’ to 3’)	Application
Swamp eel IRF10			
	Fd	GAGCGC(G)AA(G)CCAGCTGGACATC	PCR Cloning
	Rd	CTCC(G)CGCTCCAGC(T)TTGTTGGG	PCR Cloning
	R1	GCTCTTGGTGGTCACTTTCATT	5′ RACE
	R2	AAGCGGGCTGAAGAAGGTGATA	5′ RACE
	F1	AAGTGACCACCAAGAGCCCAGAT	3′ RACE
	F2	CAATGGCTCGCCTTCTTTGTCA	3′ RACE
	F3	GAAGCTGTGGAGACCGAGAG	Genomic PCR
	R3	CTGCTCTGGTGCAGTGTAGC	Genomic PCR
	F	ACAATGGCTCGCCTTCTTT	Real-time PCR
	R	TGGGACCACTCCAATACAC	Real-time PCR
Swamp eel β-actin			
	F	CAGTCCTCCTAAGGCGATAA	Real-time PCR
	R	GCATCATCTCCAGCAAAGC	Real-time PCR
Grass carp IRF10			
	Fd	ATCCCA(CG)TGGAAA(G)CACGCA(CG)GCC	PCR Cloning
	Rd	CGGGC(G)GCC(G)ACCCACAGCAGCAC	PCR Cloning
	R1	TAGAACAAACGCACCTCCAGACGG	5′ RACE
	R2	ATCTGCTTTGTCCCTGCCCTCTT	5′ RACE
	F1	GGCTGTGCTCCGGTAGGGAATG	3′ RACE
	F2	ACGGGCCTTGTGAAGCCGAGAA	3′ RACE
	F3	GAAGACAGGTCGAGGCACAT	Genomic PCR
	R3	TTATTTGGAGAAAGCAAACACA	Genomic PCR
	F	TTGACGGTGTTATGTTTTAGA	Real-time PCR
	R	CTGTAGTCCTGTTTGGCG	Real-time PCR
Grass carp β-actin			
	F	CCTTCTTGGGTAGGAGTCTTG	Real-time PCR
	R	AGAGTATTTACGCTCAGGTGGG	Real-time PCR
Trout IRF10a			
	F1	TGGTCATATTCGTTGTGCTGAAACA	PCR Cloning
	R1	CTCACCCCTCATCATGGCTG	PCR Cloning
	F	AGGTCTACCACATCCAGGCAGAGC	Real-time PCR
	R	TCCCCTGCCAAGCCCTCTCT	Real-time PCR
Trout IRF10b			
	F1	GAGGGATCATGTGATTGTGAGTAGG	PCR Cloning
	R1	CTCACCCCTCATCATGGCTG	PCR Cloning
	F	AGGCCTACCGTATCCAGACAGCAA	Real-time PCR
	R	GTGTACATGGTCCAAACCCCCACT	Real-time PCR
Trout EF-1α			
	F	CAAGGATATCCGTCGTGGCA	Real-time PCR
	R	ACAGCGAAACGACCAAGAGG	Real-time PCR

To obtain the IRF10 gene to allow analysis of exon-intron structure, genomic DNA was purified using a Wizard Genomic DNA Purification Kit (Promega, USA). Based on the full-length swamp eel and grass carp IRF10 cDNA sequences, primers (Table 1) were designed to PCR amplify the IRF10 DNA sequences. The PCR products were cloned as described below.

#### 2.2.2 Cloning of IRF10 in rainbow trout

BLAST (Basic Local Alignment Search Tool) [16] search of trout expressed sequence tag (EST) and whole-genome shotgun contigs (WGS) databases at NCBI identified a trout EST (Acc. No. EZ865213) and a WGS contig (Acc. No. MMSRT064H_scaff_1720) that appeared to encode for two IRF10 paralogues. Exons were predicted on the WGS contig and primers (Table 1) were designed for PCR amplification from a mixed cDNA sample prepared from HK macrophages stimulated independently with a set of PAMPs and recombinant trout cytokines [17]. The PCR products were ligated into pGEM-T Easy vector (Promega), transformed into *Escherichia coli* competent cells and positive clones were sequence analyzed as described previously [14, 15].

### 2.3 Sequence analysis

The nucleotide sequences generated were assembled and analyzed with the AlignIR programme (LI-COR, Inc.). The gene organization was predicted using the Spidey program at NCBI. Protein prediction was performed using software at the ExPASy Molecular Biology Server (http://expasy.pku.edu.cn/) [18]. Putative domains were identified by PROSITE (http://ca.expasy.org/prosite) [19]. Multiple sequence alignments were generated using CLUSTALW (version 1.82) [20] and shaded using BOXSHADE (version 3.21, http://www.ch.embnet.org/software/BOX_form.html). Global sequence comparisons were performed using the MatGAT program (V2.02) [21] using the scoring matrix BLOSUM62 with a gap open penalty of 10 and gap extension penalty of 1. Phylogenetic tree analysis was performed using the Maximum-likelihood method within the MEGA6.0 software [22]. The degree of confidence for each branch point was determined by bootstrap analysis (5,000 times).

### 2.4 *In vitro* transcription/translation analysis of IRF10a cDNA

**Fig 1:**
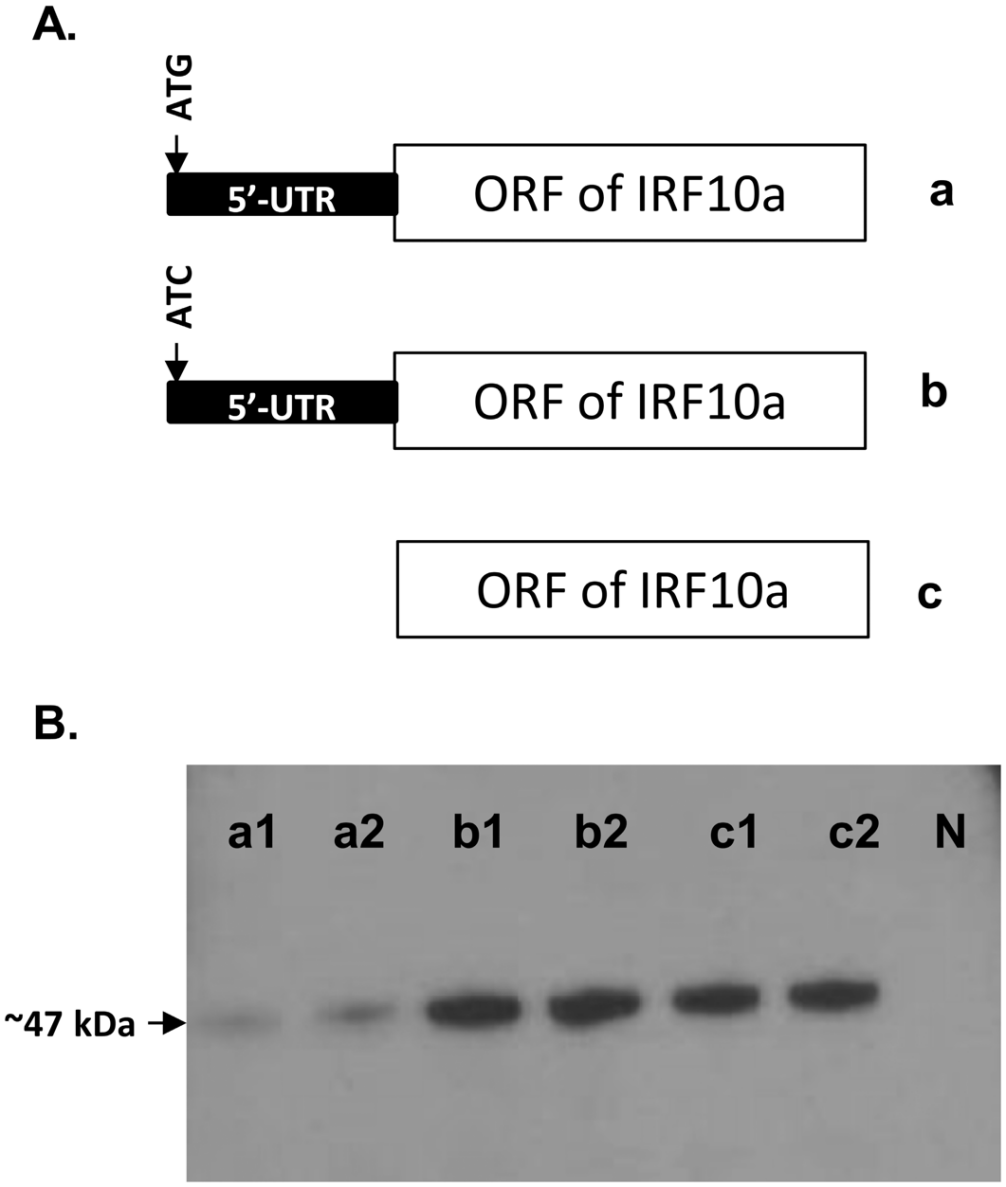
Regulation of translation by upstream ATG. **(A) Schematic diagram of the inserts of pcDNA6-IRF10a**. Three pcDNA6-IRF10a constructs were made. The construct “a” contains the 5’-UTR with the uATG and ORF of trout IFR10a. The construct “b” has the uATG mutated to ATC, and construct “c” has the 5’-UTR completely removed. **(B) Western-blot detection of translated products**. Two plasmid clones of each construct were translated using the TNT T7 Coupled Transcription/Translation and Transcend tRNA detection system. A reaction without plasmid was served as negative control (N).

To test if the upstream ATG or open reading frame (ORF) in fish IRF10 has a role in regulation of translation, the 5’-UTR and ORF of trout IRF10a was cloned downstream of the T7 promoter of the pcDNA6 vector (Invitrogen). Three constructs were made; the first contained the uATG, the second had the uATG mutated to ATC, and the third had the 5’-UTR completely removed (Fig 1A). After sequence confirmation, 1 μg of each plasmid was translated using a TNT T7 Coupled Transcription/Translation and Tanscend^™^ tRNA detection system (Promega) as per the manufacturer’s instructions. The translated proteins were labelled via biotinylated tRNA and detected by Western-blotting using Streptavidin-Alkaline Phosphatase and chemiluminescent detection, as described previously [23]. The efficiency of protein transfer was assessed by staining the membrane with Ponceau S solution (Sigma-Aldrich, UK). The protein size was determined by SeeBlue^®^ Plus2 Pre-stained Protein Standard (Life Technologies, UK).

### 2.5 Real-time PCR analysis of gene expression

For trout IRF10a and IRF10b, the primers (Table 1) for real-time PCR analysis of gene expression were designed so that at least one primer crossed an intron and were pre-tested to ensure that each primer pair could not amplify genomic DNA using the real-time PCR protocols. The expression of trout IRF10a and IRF10b, as well as the house keeping gene elongation factor-1α (EF-1α) was quantified by real-time PCR as described previously [24, 25].

For the cDNA template of swamp eel and grass carp IRF10 genes, DNase I was used to treat the total RNA before the RNA was reverse-transcribed with a cDNA Synthesis Kit (Fermentas). In this case β-actin was used as the house keeping gene to normalize the expression levels as described previously [15,26].

A standard was constructed using a mixture of equal mole amounts of purified PCR products for each gene. A serial dilution of the standards was run along with the cDNA samples in the same 96-well PCR plate and served as a reference for quantification. The expression level of each gene was calculated as arbitrary units normalized to the expression of EF-1α or β-actin. The fold changes were calculated as the average expression of the treatment groups divided by that of the relevant control group.

### 2.6 Expression of IRF10 *in vivo* in healthy fish

Six rainbow trout were killed and seventeen tissues (liver, blood, caudal kidney, spleen, heart, head kidney, skin, thymus, scales, brain, muscle, gonad, gills, tail fins, adipose tissue, adipose fin and intestine) collected for RNA extraction using TRI Reagent (Applied Biosciences) following the manufacturer’s instructions. The real-time PCR analysis was as described above.

Major immune tissues/organs were also collected from four Asian swamp eels or grass carp and IRF10 expression analyzed. Since these three species, Asian swamp eel, grass carp and rainbow trout, belong to three different teleost orders, i.e. the Synbranchiformes, Cypriniformes and Salmoniformes, respectively, with different physical characteristics, an identical set of tissues for all the species was not possible. For example, the adipose fin is absent in Asian swamp eel and grass carp, and scales are absent and gills poorly developed in Asian swamp eel. Thus, fewer types of tissues were analyzed in Asian swamp eel and grass carp.

### 2.7 Modulation of trout IRF10 paralogues *in vitro*

Primary HK macrophage cultures were prepared from four individual fish, as outlined by Costa et al. [23]. At day 4 primary macrophages were stimulated with Poly I:C (50 μg/ml, Sigma), peptidoglycan (PGN, 5 μg/ml, InvivoGen), trout recombinant (r) IL-1β (20 ng/ml) [27], rIL-6 (100 ng/ml) [23], rTNF-a3 (10 ng/ml) [17], rIFNγ (20 ng/ml) [28], rIL-12A (p35a1/p40c) and rIL-12B (p35a1/p40b1) [29, 30] for 4 h, 8 h and 24 h. RNA extraction and real-time PCR analysis was conducted as described above. Although crude LPS is a potent inducer of immune gene expression in rainbow trout macrophages, pure LPS alone has little effect [17, 31], thus LPS stimulation was excluded from the current analysis.

### 2.8 Modulation of swamp eel IRF10 expression *in vivo* by Poly I:C

Two groups of 12 swamp eels were injected intraperitoneally (i.p.) with either 0.5 ml Poly I:C in phosphate buffered saline (PBS), at 2 mg/ml, or 0.5 ml PBS to serve as a control. Four eels from each group were killed at 12 h, 24 h, and 48 h post-injection, and HK and spleen were collected for total RNA extraction and gene expression analysis as above.

### 2.9 Modulation of grass carp IRF10 expression *in vivo* by grass carp haemorrhagic virus (GCHV)

Grass carp fingerlings were injected i.p. with 0.5 ml GCHV (4×10^8^ tissue culture infectious dose (TCID)_50_/ml) [32]. Control groups were injected with 0.5 ml PBS. Four fish from each group were sampled on days 1 to 7 post injection. Total RNA from HK and spleen samples was extracted using Trizol reagent (Invitrogen), and real-time PCR analysis conducted as described above.

### 2.10 Modulation of trout IRF10 paralogue expression *in vivo* by viral haemorrhagic septicaemia virus (VHSV)

This experiment used archived tissue samples from an experiment reported previously [33]. In brief, pathogenic VHSV DK-F1 or the non-pathogenic strain DK-M.Rhabdo was propagated at 15°C on 90% confluent monolayers of BF-2 cells (bluegill fry, ATCC No. CCL-91) cultured in L-15 supplemented with 5% FCS and 1% L-glutamine (200mM). Once full cytopathic effects (CPE) were observed, the virus was stored at -80°C and a thawed aliquot titrated on BF-2 cells by end point dilution. Before challenge, the fish was screened and confirmed to be free of VHSV, infectious pancreatic necrosis (IPNV), proliferative kidney disease (PKD), enteric redmouth disease (ERM), bacterial kidney disease (BKD), salmonid alphavirus (SAV) and furunculosis [34]. For the challenge, fish were injected i.p. with 100 μl DK-F1 (1 × 10^8^ 50% tissue culture infective dose (TCID50)/fish) or DK-M.Rhabdo (1 × 10^8^ TCID50/fish) or media as control. Four fish from each group were killed and kidney collected at 1, 2, 3, 4, 5, 7 and 9 days post-infection. Real-time PCR analysis of IFR10 expression was conducted as described above.

### 2.11 Statistical analysis

The SPSS package 20.0 was used for statistical analysis of changes of gene expression levels. A fold change was calculated as described previously [15]. To improve the normality of real-time quantitative PCR measurements, a log2 transformation of the arbitrary units after normalization to the level of EF-1α expression was performed as described previously [24]. A Paired-Sample T-test was applied to compare the expression levels of trout IRF10 paralogues *in vivo* and *in vitro* after stimulation. One way-analysis of variance (ANOVA) followed by the LSD post hoc test (when appropriate) were used to analyze the *in vivo* expression data, with P <0.05 between treatment groups and control groups considered significant.

## Results

### 3.1 Molecular characterization of IRF10 in three fish species

An IRF10 cDNA sequence was isolated from grass carp and Asia swamp eel (S1 and S2 Figs), and two IRF10 paralogues from rainbow trout (S2 Fig). The grass carp IRF10 cDNA (Acc. No: FJ556996) is 1,501 bp long and contains an open reading frame (ORF) of 1194 bp encoding for 397 aa, a 5'-untranslated region (UTR) of 138 bp and a 3'-UTR of 169 bp (S1 Fig). Two in frame stop codons and four potential upstream (u) ORFs are present in the 5’-UTR before the main ORF. There are three potential polyadenylation signals (AATAAA) in the 3'-UTR with the last one 12 bp upstream of the poly (A) tail of the grass carp IRF10 cDNA. In addition, there is an mRNA instability motif (ATTTA) in the 3’-UTR (S1 Fig).

The swamp eel IRF10 cDNA (Acc. No: KM213622) is 1,744 bp long and contains an ORF of 1236 bp encoding for 411 aa, a 5'-UTR of 60 bp and a 3'-UTR of 428 bp. A potential uORF is present in the 5’-UTR before the main ORF. There are two mRNA instability motifs and a polyadenylation signal 21 bp upstream of the poly (A) tail in the 3'-UTR of the swamp eel IRF10 cDNA (S2 Fig).

The trout IRF10a (Accession No: HG917960) and IRF10b (Accession No: HG917961) have ORFs of 1254 bp and 1164 bp encoding for 417 aa and 387 aa, respectively (S3 Fig). There are in frame stop codons in the 5’-UTR before the main ORFs of the trout IRF10 paralogues, suggesting the ORFs are complete. Two in frame uATGs are present in the 5’-UTR of trout IRF10a, that when translated extend the uORF of 255 bp into the main ORF. A uORF is also present in the 5’-UTR of the trout IRF10b cDNA (S3 Fig). The cDNA sequences of trout IRF10a and IRF10b share 88% identity in the coding region.

To test if the uATG or uORF has a role in regulation of translation in fish, the 5’-UTR and ORF of trout IRF10a was cloned to a pcDNA6 vector with/without the first uATG mutated or with the 5’-UTR completely removed (Fig 1A). Coupled transcription/translation analysis revealed that all the constructs can be translated to a protein of the expected size (~47 kDa). However, the translated protein was reduced in the presence of the uATG (Fig 1B). No bands could be detected in the control with no plasmids and specific band could be detected with plasmids with different inserts (data not shown).

At the protein level, trout IRF10a and IRF10b have 77.7% identity to each other. The grass carp IRF10 has highest identity to zebrafish IRF10 (75.0%) and swamp eel IRF10 has highest identity to Japanese flounder IRF10 (73.2%) (Table 2). The teleost IRF10 molecules share higher identities between each other (49.5–77.7%) than to tetrapod IRF10s (40.9–47.8%). Of the nine other tetrapod IRF members known, fish IRF10 molecules have highest identities to IRF4 (38.0–42.8%), followed by IRF8 (36.3–40.7%) and IRF9 (31.2–35.7%) of the IRF4 subfamily (Table 2). They have only low identities to the IRF5/6 subfamily (27.2–30.4%), the IRF3/7 subfamily (23.0–27.1%) and the IRF1/2 subfamily (20.6–26.2%) (Table 2).

**Table 2:** Identity comparison between teleost and homeotherm IRF10 molecules, and fish IRF10 and selected tetrapod IRF molecules.

			Identity (%)					
Molecule	Species	Amino acids	Trout-a	Trout-b	Swamp eel	Grass carp	Flounder	Zebrafish
IRF10								
	Trout-a	*417*		77.7	55.1	58.6	57.5	55.9
	Trout-b	*387*	77.7		57.0	58.4	58.5	54.5
	Swamp eel	*411*	55.1	57.0		52.6	73.2	49.5
	Grass carp	*397*	58.6	58.4	52.6		54.5	75.0
	Flounder	*404*	57.5	58.5	73.2	54.5		52.5
	Zebrafish	*392*	55.9	54.5	49.5	75.0	52.5	
	Cow	*398*	43.5	44.9	42.8	45.6	43.0	43.8
	Bat	*435*	42.2	41.9	40.9	44.5	42.7	42.9
	Chicken	*416*	47.8	46.8	44.9	46.5	47.3	45.2
IRF4								
	Human	*451*	40.2	40.1	38.0	42.0	38.4	38.6
	Mouse	*450*	39.5	40.6	38.2	42.8	38.5	39.4
	Chicken	*445*	42.0	41.0	39.6	43.0	38.7	40.6
IRF8								
	Human	*426*	38.9	37.9	36.7	40.5	36.5	39.1
	Mouse	*424*	39.2	38.6	36.9	39.7	36.0	40.0
	Chicken	*425*	38.0	36.3	36.3	39.5	36.4	40.7
IRF9								
	Human	*393*	34.6	32.0	32.0	35.7	32.5	35.7
	Mouse	*399*	33.2	31.4	31.2	33.8	32.0	34.2
IRF1								
	Human	*325*	23.7	25.5	22.7	24.3	22.3	26.0
	Mouse	*329*	25.1	26.2	22.4	24.8	21.8	26.9
	Chicken	*313*	23.9	24.6	21.9	22.4	20.6	21.3
IRF2								
	Human	*349*	22.4	22.6	21.1	26.4	20.7	23.9
	Mouse	*349*	23.1	22.8	22.5	26.3	22.8	25.7
	Chicken	*348*	24.1	24.9	22.1	24.8	24.4	25.4
IRF3								
	Human	*427*	26.3	23.7	23.0	26.3	26.5	26.3
	Mouse	*419*	24.4	22.9	23.8	26.2	26.5	25.6
IRF7								
	Human	*503*	24.3	22.6	26.3	24.8	24.7	25.0
	Mouse	*457*	25.8	25.2	27.1	24.0	26.2	23.7
	Chicken	*491*	24.5	24.4	24.1	26.3	24.7	25.0
IRF5								
	Human	*498*	29.7	27.6	27.2	28.8	27.5	29.5
	Mouse	*497*	28.4	27.9	28.3	29.4	28.5	28.8
	Chicken	*472*	30.1	28.1	27.8	28.9	29.1	30.4
IRF6								
	Human	*467*	30.2	27.6	27.8	29.6	29.6	29.1
	Mouse	*467*	30.0	28.3	28.5	30.2	30.0	29.1
	Chicken	*470*	29.9	28.8	30.3	29.7	29.9	29.2

**Fig 2:**
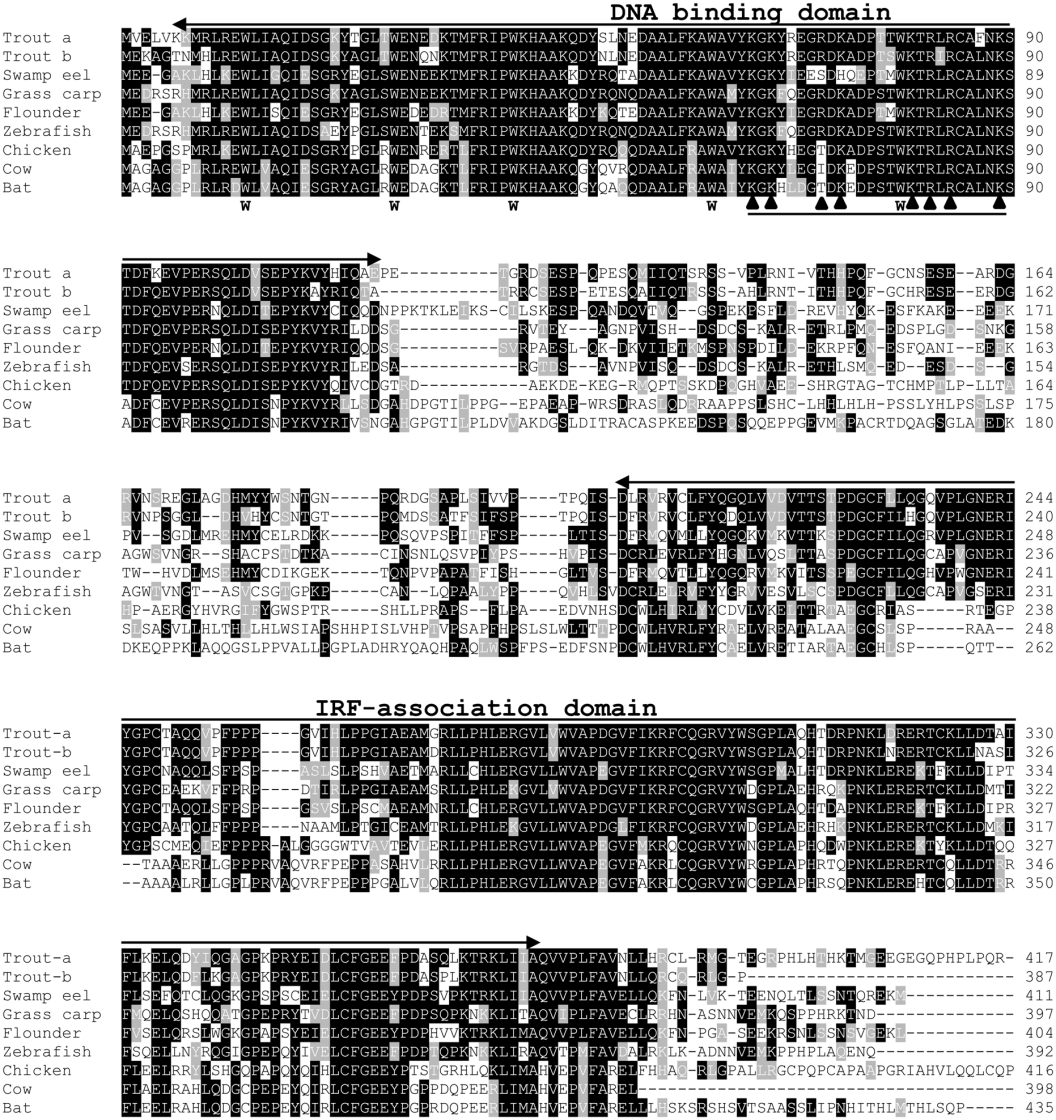
Multiple alignment of IRF10 molecules from rainbow trout, grass carp and swamp eel with selected IRF10 from other fish, birds and mammals. Dashes (-) indicate gaps introduced into the alignment and conserved amino acids are shaded. The DNA-binding domain and IRF association domain are denoted above the alignment. The tryptophan (W) residues of the five W-rich repeats and basic amino acids in the nuclear localization signal are indicated below the alignment with Ws and up headed arrows, respectively. The accession numbers for IRF10 sequences used in this alignment are D2KTW2 (flounder), A8E564 (zebrafish), Q90WI0 (chicken), E1BKD9 (cow) and L5JZZ9 (bat).

A multiple alignment of teleost IRF10 with IRF10 molecules from birds and mammals revealed well conserved DBD and IAD domains (Fig 2). The five tryptophans that form the five conserved W-rich repeats are completely conserved in the DBD. A potential nuclear localization signal with multiple basic amino acids (R/K) [35] is also present in the DBD. The middle region linking the DBD and IAD, and the C-terminal end are less conserved (Fig 2).

To further investigate the relationship between teleost IRF10 molecules with other vertebrate members of the IRF family, a phylogenetic tree was constructed using the Maximum likelihood method and bootstrapped 5,000 times. The IRF10 molecules from teleost fish species and from tetrapods formed two independent clades, grouped together with high bootstrap support (99%) and separated from other vertebrate IRF members, confirming their identities (S4 Fig). Consistent with the homology analysis (Table 2), IRF10 and IRF4 grouped together first and clustered with IRF8 and IRF9, that define the IRF4/8/9/10 subfamily. The teleost IRF11 molecules clustered with vertebrate IRF1 and IRF2, that may define an extended IRF1/2/11 subfamily (S4 Fig).

### 3.2 Gene organization of teleost IRF10

**Fig 3:**
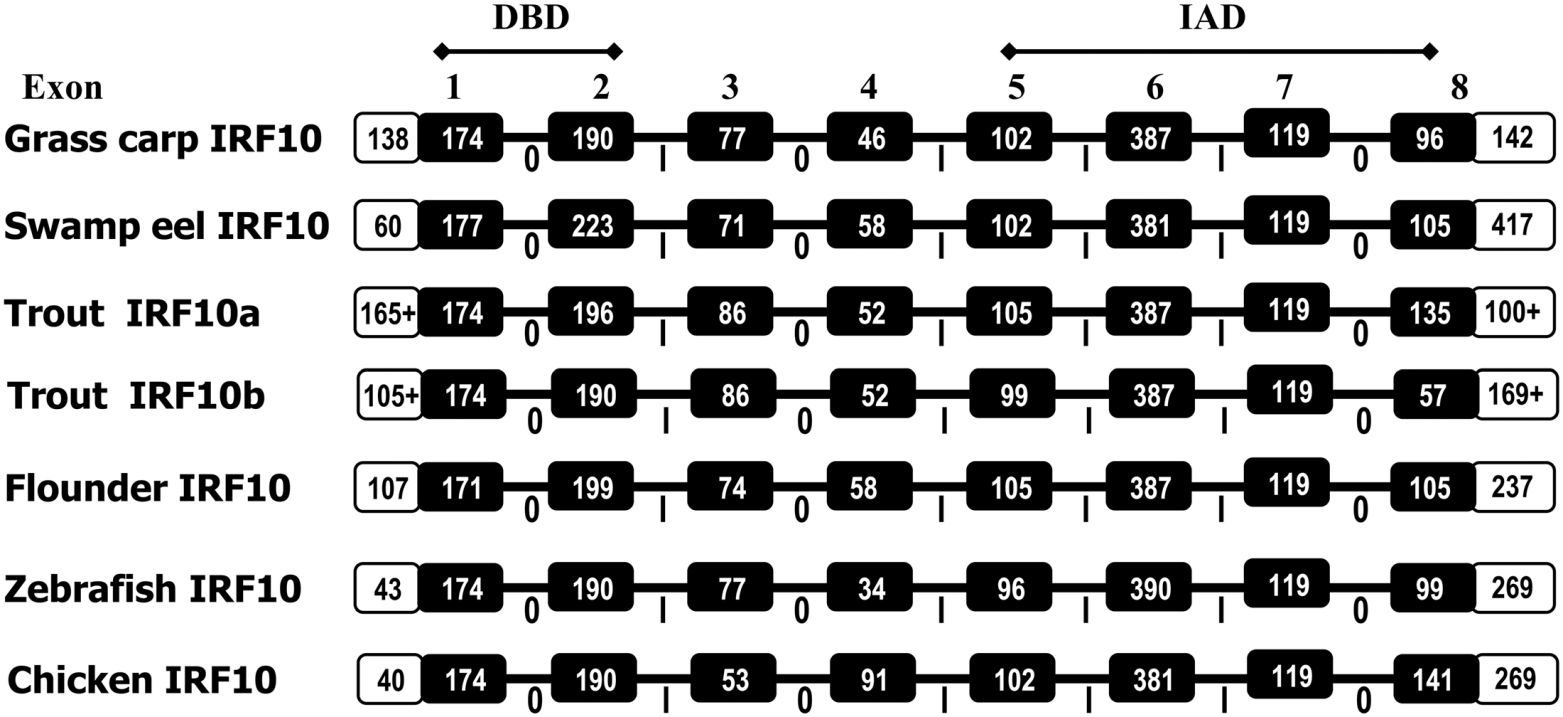
Schematic diagrams of exon-intron structure of IRF10 genes. Boxes represent exons and horizontal lines connecting exons represent introns. Open reading frames and untranslated regions are shown as black boxes and white boxes respectively. The number in the box is the nucleotide length (base pair). The intron phase is indicated under the bar. The grass carp (acc. no. FJ556997) and swamp eel (acc. no. KM213622) IRF10 genes were sequenced in this report. The gene organization of trout IRF10a and IRF10b was predicted from a WGS contig (Acc. No. MMSRT064H_scaff_1720). The flounder IRF10 was reported by Suzuki et al., (2011). The chicken and zebrafish IRF10 gene organization was derived from Ensembl genes ENSGALG00000006448 and ENSDARG00000027658, respectively.

The gene organization of IRF10 was predicted previously from genome sequence information with inconsistent exon and intron numbers in different species [7]. We clarified this issue by cloning the IRF10 genomic sequence from grass carp and swamp eel. It appears that the fish and bird IRF10 genes have a general 8 exon/7 intron gene organization with introns 1, 3 and 7 in phase 0 and the rest of the introns in phase I. The sizes of first two exons that encode for the DBD and exons 5–7 that encode for the IAD were relatively well conserved in different species. However, exons 3 and 4 that encode for the region between the DBD and IAD, and the final exon that encodes the C-terminal were more variable between different species (Fig 3).

### 3.3 Tissue distribution of the expression of IRF10 paralogues in rainbow trout

**Fig 4:**
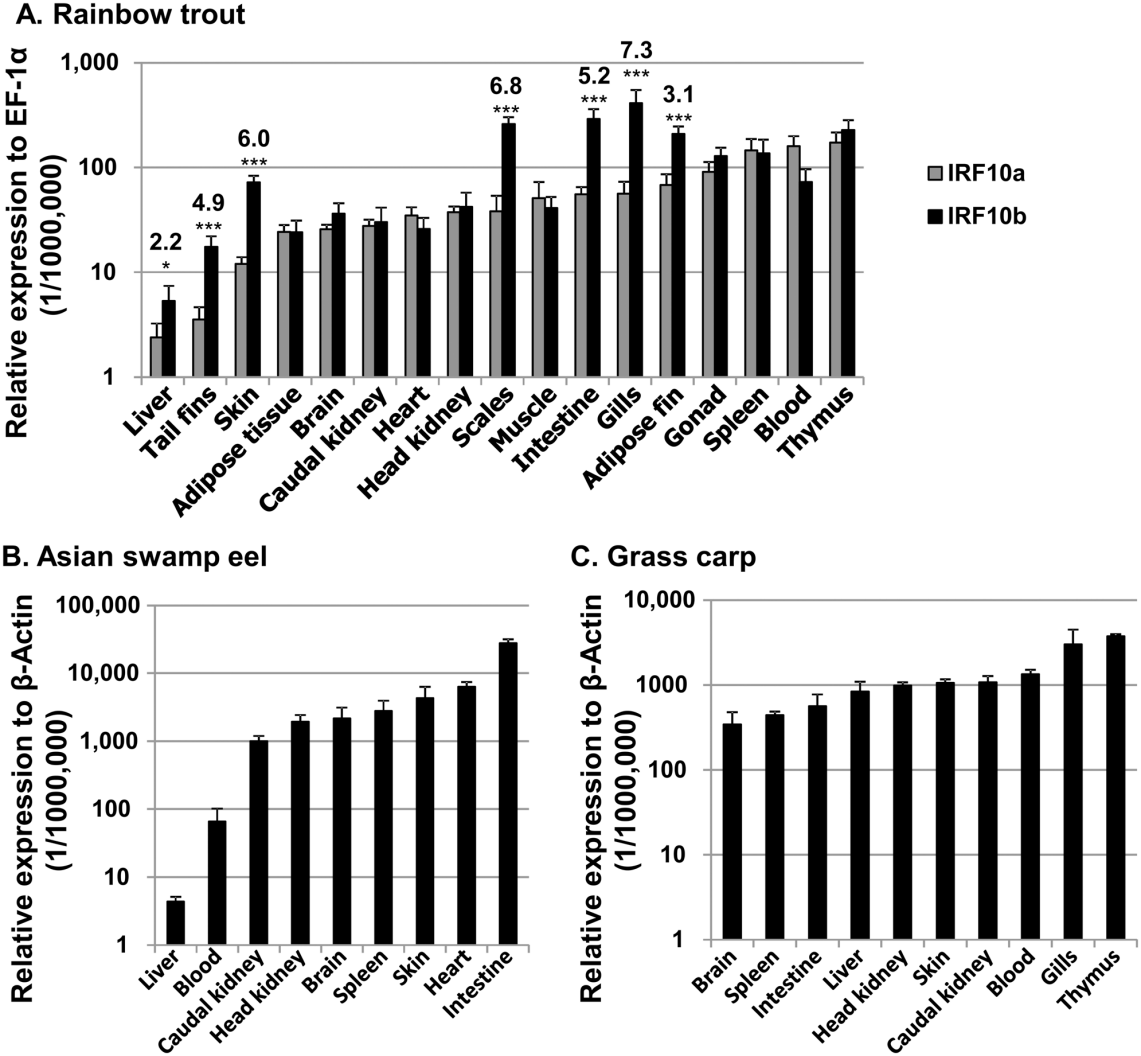
Constitutive expression of IRF10 *in vivo* in (A) rainbow trout, (B) Asian swamp eel and (C) grass carp. **(A)** The transcript expression level of rainbow trout IRF10 paralogues was determined by real time RT-PCR in 17 tissues from six rainbow trout. The expression level for each gene is presented relative to the expression level of EF-1α. The results represent the average + SEM of six fish. The p value of paired sample t-tests is shown as: *p≤0.05 and ***p≤0.001. The numbers above the stars are the ratio of the expression levels of IRF10b vs IRF10a. **(B)** and **(C)**, IRF10 expression in Asian swamp eel and grass carp was determined as in rainbow trout but presented relative to the expression level of β-actin. The results represent the average + SEM of four fish.

The expression of the trout IRF10 paralogues was comparatively examined in seventeen tissues from six healthy trout, using an equal molar reference in the same real-time PCR plate. The expression of both genes was detectable in all tissues examined, with liver expressing the lowest level of both IRF10a and IRF10b (Fig 4A). The highest expression of IRF10a was in thymus, blood and spleen, whereas the highest expression of IRF10b was in gills, intestine, scales, adipose fin and thymus. Most of the tissues express similar levels of IRF10a and IRF10b except in the integumentary tissues (gills, scales, skin, intestine, tail fins and adipose fin), where the expression of IRF10b was 7.3-, 6.8-, 6.0-, 5.2-, 4.9- and 3.1-fold higher than that of IRF10a, respectively. The expression of trout IRF10b was also higher than that of IRF10a in liver (Fig 4A).

The expression of IRF10 orthologues was also examined in several tissues of healthy Asian swamp eel and grass carp. The highest IRF10 expression was detected in intestine and lowest in liver in Asian swamp eel (Fig 4B). High levels of IRF10 were also detected in heart, skin, spleen, brain HK and caudal kidney in the eel (Fig 4B). The grass carp IRF10 expression was high in all the tissues examined, with the highest expression in thymus and gills (Fig 4C).

### 3.4 Differential expression and modulation of trout IRF10 paralogues *in vitro*

**Fig 5:**
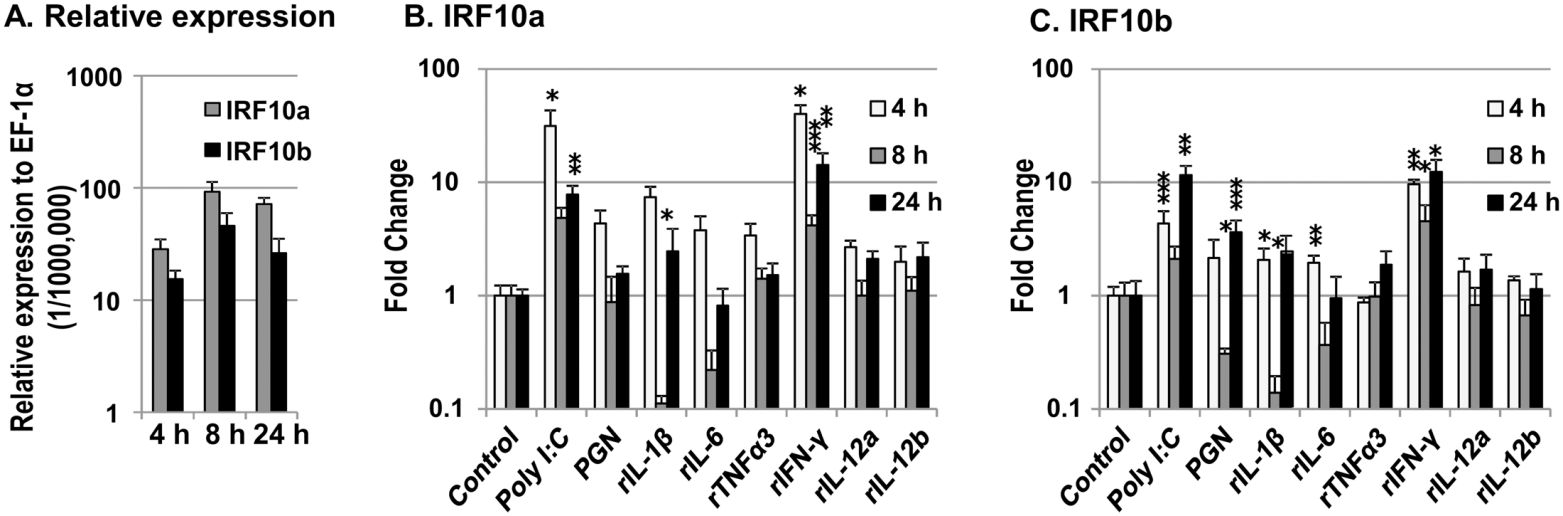
Modulation of expression of trout IRF10a and IRF10b in primary HK macrophages. The relative expression levels in the control cells are shown in (A). The primary HK macrophages were stimulated with Poly I:C (50 μg/ml), peptidoglycan (PGN, 5 μg/ml) rIL-1β (20 ng/ml), rIL-6 (200 ng/ml), rTNFα3 (10 ng/ml), rIFN-γ (20 ng/ml), rIL-12A (p35a1/p40c) and rIL-12B (p35a1/p40b1) for 4 h, 8 h and 24 h. The fold changes of IRF10a (B) and IRF10b (C) were calculated as the average expression level of stimulated samples divided by that of the time-matched controls. The means+SEM of four fish are shown. The p-values of paired samples T tests between stimulated samples and their time matched controls is shown above the bars as:*p≤ 0.05, **p≤0.01 and ***p≤0.001.

Macrophages are specialized phagocytic cells that attack foreign substances, pathogens and cancer cells, function in innate immunity and help to initiate adaptive immunity in vertebrates. Thus we investigated the modulation of the two trout IRF10 genes in primary macrophage cultures after stimulation with PAMPs and recombinant trout cytokines. The expression of both trout IRF10a and IRF10b were detectable at similar low levels during the stimulation period (Fig 5A). Poly I:C and rIFN-γ were strong inducers of the expression of both trout IRF10 paralogues with a two-phase induction seen (Fig 5B and 5C). Poly I:C induced IRF10a expression peaked at the early time point (22.4-fold at 4 h), dropped back to the control level at 8 h, and began to increase again at the late time point (9.0-fold at 24 h). Similarly trout IRF10b expression was also induced at 4 h (5.1-fold) and 24 h (11.7-fold), with no significant induction at 8 h. rIFN-γ significantly induced both IRF10a and IRF10b expression at 4 h, 8h and 24 h, with a fold change of 20.7, 4.5 and 18.0 for IRF10a, and 8.8, 7.1 and 15.0 for IRF10b, respectively.

Peptidoglycan is a polymer consisting of sugars and amino acids and is a major component of the bacterial cell wall. It induces the expression of pro-inflammatory cytokines, e.g. IL-1β, TNF-α and IL-6 in trout macrophages [16, 31]. Peptidoglycan had no effect on trout IRF10a expression, however it significantly decreased IRF10b expression at 8h, but increased its expression at 24 h (3.4-fold) (Fig 5B and 5C).

The pro-inflammatory cytokines IL-1β and IL-6 induced a modest early induction of IRF10b expression at 4 h but the expression of both IRF10a and IRF10b was significantly inhibited at 8 h by IL-1β stimulation. Both genes were refractory to stimulation by TNF-α3, IL-12a (p35a1/p40c) and IL-12b (p35a1/p40b1) (Fig 5B and 5C).

### 3.5 Modulation of IRF10 expression *in vivo* by Poly I:C and viral infection

**Fig 6:**
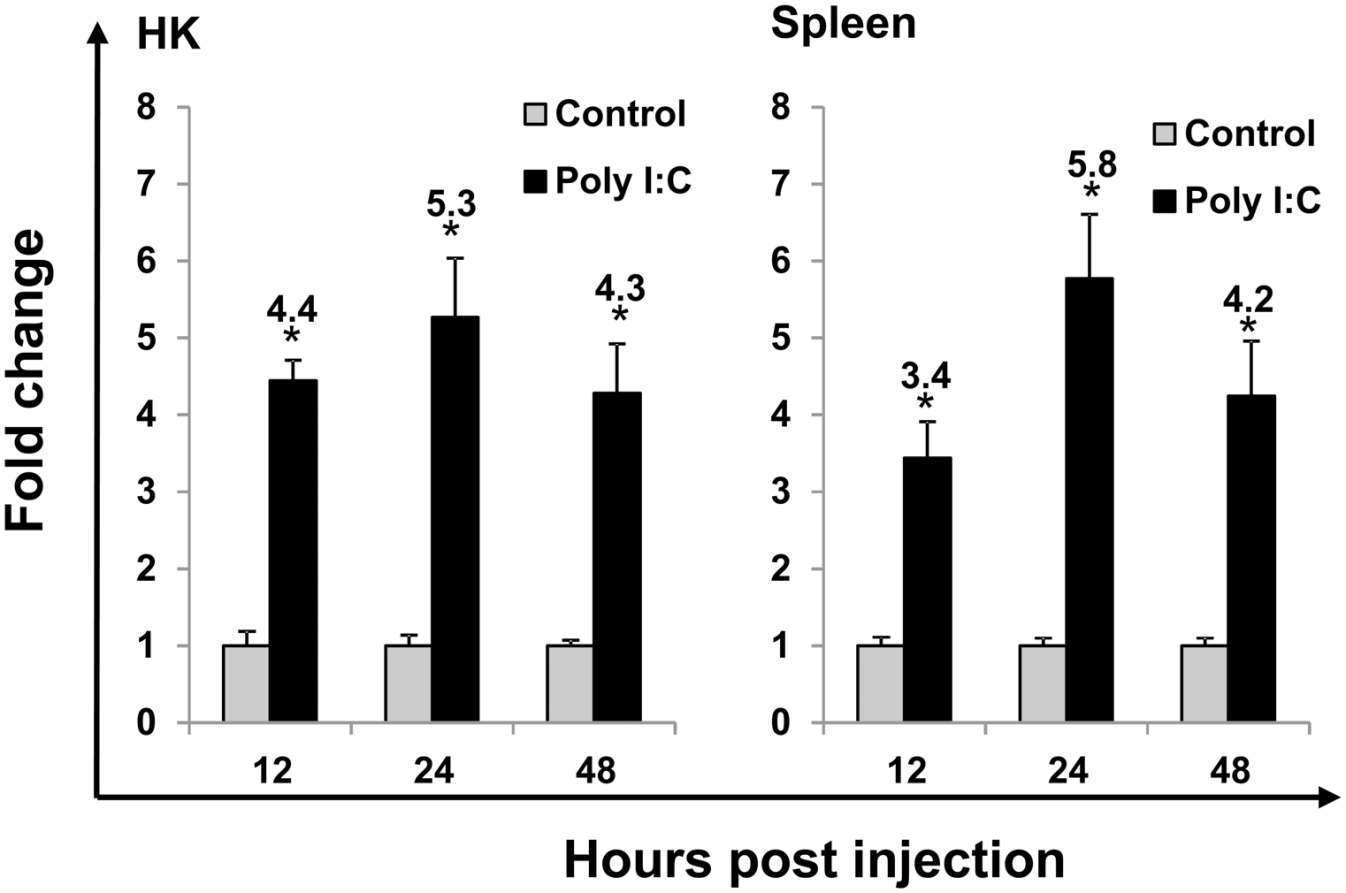
Induction of Asian swamp eel IRF10 expression by Poly I:C in HK and spleen. Swamp eel were injected i.p. with either Poly I:C, or PBS to serve as a control. HK and spleen tissues were collected at 12 h, 24 h, 48 h for total RNA extraction and gene expression analysis by real-time PCR. The means+SEM of four fish are shown. The relative significance of a LSD post hoc test after a significant one way-ANOVA between the Poly I:C and PBS injected groups at the same time point is shown above the bars as: *p<0.05. The numbers above the bars are the fold changes, calculated as the average expression level of stimulated samples divided by that of the time-matched controls.

Poly I:C was a potent inducer of trout IRF10 expression *in vitro*. Thus, we further investigated its impact on IRF10 expression *in vivo* in swamp eel. The expression of swamp eel IRF10 was significantly increased from 12 h to 48 h in both HK (up to 5.3-fold) and spleen (up to 5.8-fold) (Fig 6).

**Fig 7:**
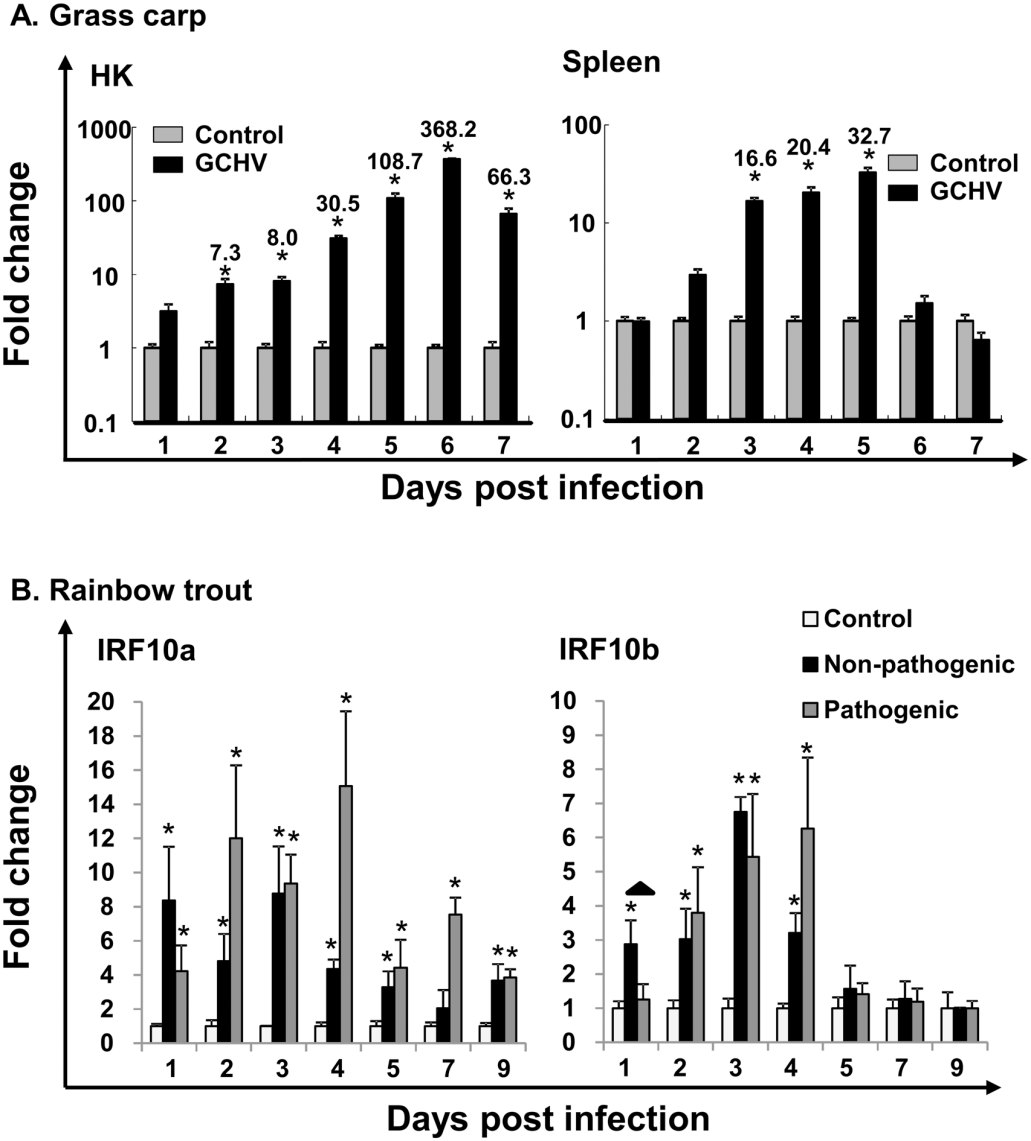
Modulation of IRF10 expression grass carp (A) and rainbow trout (B). **(A)** Healthy grass carp fingerlings were injected i.p. with GCHV (2×10^8^ TCID50/fish). Control groups were injected with PBS. Four fish were sampled daily to day 7 and gene expression analyzed as in Fig 6. The means+SEM of four fish are shown. The relative significance of a LSD post hoc test after a significant one way-ANOVA between the GCHV and PBS injected groups at the same time point is shown above the bars as: *p<0.05. The numbers above the bars are the fold changes, calculated as the average expression level of infected samples divided by that of the time-matched controls. **(B)** Rainbow trout were injected i.p. with pathogenic (DK-F1, 1 × 10^8^ TCID50/fish) or non-pathogenic (DK-M.Rhabdo, 1 × 10^8^ TCID50/fish) strains of VHSV, or control media as control. Head kidney tissue was collected at days 1, 2, 3, 4, 5, 7 and 9 after challenge, and expression analysis performed as above. The expression of the IRF10 paralogues was first normalised to that of EF-1α and then expressed as a fold change calculated as the average expression level of viral infected samples divided by that of the time-matched controls. Results are means + SEM (n = 4). Asterisks indicate significant differences (P < 0.05) relative to time-matched control. The up arrow shows significant difference (p<0.05) between pathogenic and non-pathogenic VHSV infected samples.

Poly I:C is a (synthetic) double-stranded (ds)RNA that is known to be present in some viruses, e.g. Reoviruses. The induction of IRF10 expression *in vitro* and *in vivo* by Poly I:C prompted the further study of IRF10 expression in grass carp infected by GCHV and in rainbow trout by VHSV. There is no viral infection model available for Asian swamp eel. GCHV, is a member of the Reoviridae that contains a genome composed of 11 dsRNA segments, and causes a severe hemorrhagic disease of grass carp in China [32]. Grass carp IRF10 expression was significantly increased in HK from day 2 to day 7 post-infection with GCHV, with a fold increase peaking at day 6 (368.2-fold). Its expression was also up-regulated (up to 32.7-fold) in spleen from day 3 to day 5 after GCHV infection (Fig 7A).

Viral haemorrhagic septicaemia caused by VHSV is one of the most important viral diseases of salmonid fish in European aquaculture. VHSV has been isolated from more than 60 fish species, with rainbow trout the most susceptible [36]. The kidney is one of the major targets of VHSV infection, and thus the expression of IRF10 paralogues was examined in this tissue. The impact on IRF10 expression of infection with a pathogenic and non-pathogenic VHSV was investigated. The expression of trout IRF10a was significantly increased from day 1 to day 9 after infection by pathogenic VHSV and from day 1 to day 5 and day 9 by nonpathogenic VHSV (Fig 7B). The IRF10b expression was also increased from day 1 to day 4 by non-pathogenic VHSV and from day 2 to day 4 by pathogenic VHSV (Fig 7B). It is noteworthy that at day 1, IRF10b expression was induced and significantly higher (p<0.05) in fish infected by non-pathogenic VHSV than by pathogenic VHSV (Fig 7B).

## Discussion

Absent in humans and mice, the functional role of IRF10 in vertebrate immunity is relatively unknown compared to IRF1-9. The cloning of four IRF10 genes in three diverse and economically important fish species in this report will facilitate future evaluation of this molecule in fish innate and adaptive immunity.

### 4.1 Molecular characteristics of fish IRF10

IRF10 was first reported in chicken [10]. The identity of fish IRF10 molecules, including the four described in this report was supported by (1) higher identities to tetrapod IRF10 molecules than to any other vertebrate IRF members (Table 2); (2) well conserved DBD and IAD domains relative to IRF10 molecules from different vertebrates (Fig 2); (3) clustering of the fish IRF10 molecules with tetrapod IRF10 with 99% bootstrap support in the phylogenetic tree (S4 Fig); (4) a conserved gene organization of the fish and bird IRF10 (Fig 3); and (5) conserved gene synteny in the IRF10 loci of mammals, birds, reptiles, amphibians and fish [7].

#### IRF10 is most closely related to IRF4 in the IRF4 subfamily

The vertebrate IRF family is further divided into 4 subfamilies, the IRF1, IRF3, IRF4 and IRF5 subfamilies that consist of IRF1/IRF2, IRF3/IRF7, IRF4/IRF8/IRF9, and IRF5/IRF6, respectively [10]. The grouping of IRF10 in the IRF4 family was not well resolved in the phylogenetic tree of Suzuki et al. [12]. Our Maximum likelihood tree clearly grouped IRF10 to IRF4 first, and then to IRF8 and IRF9 within the IRF4 subfamily (S4 Fig). Our tree was further supported by homology analysis (Table 2), where IRF10 sequences have higher identities to IRF4 than to IRF8 and IRF9. A similar tree topology has also been reported by Stein et al. [6] and Huang et al. [7]. Thus the closest relative of IRF10 is IRF4.

#### IRF10 in fish and birds has an 8 exon/7 intron structure

Previous analysis of sequenced genomes predicted that IRF10 genes have 6–11 exons in different vertebrates [7]. Our analysis of IRF10 gene structure in three further fish species revealed a general 8 exon/7 intron gene organization in fish and birds. The sizes of the exons encoding for the DBD and IAD domains are well conserved, as are the intron phases (Fig 3). The gene organization still needs to be confirmed by sequencing of transcripts in other species.

#### Regulation of fish IRF10 by uORF/uATG and mRNA stability

In eukaryotic mRNA the main ORF is flanked by upstream and downstream regulatory regions of variable length and structure. These regions may contain multiple regulatory cis-acting sequence elements, including uAUGs or uORFs in the 5’-UTR, and AUUUA motifs in the 3’-UTR [37]. There are four uORF in grass carp IRF10, and one each in the 5’-UTR of trout IRF10b, swamp eel IRF10 and flounder IRF10 [12]. Furthermore, two in frame uATGs are present in the 5’-UTR of trout IRF10a, that when translated extend the uORF into the main ORF (S3 Fig). According to the scanning model, 40S ribosomal subunits are recruited to the 5’-terminal cap structure, scan in the 5’ to 3’-direction, and can initiate translation at the first AUG they encounter. Translation of a downstream ORF is possible by either leaky scanning or re-initiation [38]. Thus, uAUGs that constitute the initiation codon of uORFs, interfere with unrestrained ribosomal scanning toward the main ORF initiation codon. The presence of uORFs in IRF10 of other species is unclear because of incomplete sequence information. The presence of uATGs/uORFs in all the four IFR10 genes reported here and in flounder IRF10 may hint at multiple regulatory mechanisms at the translational and post-translational levels, perhaps required to regulate IRF10 function. The regulatory effect of uATG has been demonstrated in rainbow trout IRF10a by an *in vitro* transcription/translation system (Fig 1), but would still need to be validated in fish cells.

Post-transcriptional regulation plays a vital role in controlling the expression of cytokines by modulating mRNA stability mediated by AU-rich elements (e.g. ATTTA motifs, or AREs) located in the 3'-UTR [39]. These AU-rich elements are also present in the 3’-UTR in many fish cytokines e.g. IL-1β and TNF-α [17], and have a role in post-transcriptional regulation of fish cytokines [40]. The presence of one ATTTA motif in grass carp IRF10 and two in swamp eel suggests that IRF10 mRNA may also be regulated by this mechanism at the post-transcriptional level [39–40].

#### Two IRF10 genes in rainbow trout

A single IRF10 gene exists in other fish species and tetrapods [12] but two are present in rainbow trout. The trout IRF10 paralogues share highest identity amongst the IRF10 molecules from different species (Table 2), suggesting that they may have arisen from the salmonid whole genome duplication event as seen in other salmonid paralogous genes [16, 41– 43]. The coding region of the trout IRF10 nucleotide sequences have 88% identity but share only 41.3% and 44.1% identity in the known 5'-UTR and 3'-UTR, respectively. This may hint at differential regulation at the post transcriptional level. One difference is the number of uAUG in the 5’-UTR. In trout IRF10b, the uAUG may function as the start codon of the only uORF, which has a stop codon before the main ORF. Thus, the full-length IRF10b can be translated by either leaky scanning or re-initiation [38]. There are two in frame uAUGs present in the 5’-UTR of trout IRF10a. A uORF that starts translation from either uAUG will extend into the main ORF. Thus the full-length IRF10a can only be translated by leaky scanning. Re-initiation of translation of the main ORF after the uORF will produce a truncated IRF10a protein isoform starting from M132 that lacks the DBD essential for DNA binding. Such a truncated IRF10a may function as a negative regulator of full-length IRF10a or IRF10b. Our functional studies here provide evidence that the presence of the uATG/uORF dampens trout IRF10a translation, confirming this mechanism of regulation *in vitro*.

### 4.2 Tissue distribution of the expression of IRF10

Although direct comparison of IRF10 expression was difficult between species in this study, nevertheless common expression patterns were present. For example, high levels of IRF10 expression were observed in fish tissues expected to be rich in T cells, such as the thymus and gills, in both rainbow trout and grass carp (Fig 4), suggesting a potential role in fish T cell development. Large differences in expression levels were apparent in different tissues, with lowest levels in liver in both rainbow trout and Asian swamp eel. Some species-specific expression patterns were also seen. The intestine of Asian swamp eel had the highest level of IRF10 in all the tissues examined. Trout intestine was also one of the tissues with the highest level of IRF10b expression. However, grass carp intestine expressed only moderate levels of IRF10. Intestinal IRF10 expression may be linked to the feeding habits of different fish species. Grass carp feed primarily on aquatic plants, whilst Asian swamp eels are voracious predators and eat fish, eggs, frogs, shrimps and other aquatic invertebrates [44]. Rainbow trout are also predators with a varied diet. The intestine of a predator will be at higher risk of food-borne infections, especially viral infections, than that of an herbivorous fish. Thus the high levels of IRF10 expression in predatory species may be linked to the need to combat food-borne disease.

Differential expression of paralogous genes can suggest potential subfunctionalisation [42]. The trout IRF10 paralogues are expressed at similar levels in most of the internal tissues examined. However, their expression in integumentary tissues was significantly different. IRF10b expression was significantly higher than IRF10a in gills, scales, skin, intestine, adipose fin and tail fins, components of fish mucosal immunity and potential entry sites of waterborne pathogens [45]. Thus trout IRF10b may be more important in mucosal immunity.

### 4.3 Modulation of trout IRF10 expression by PAMPs and recombinant cytokines

Macrophages are important for immune responses to pathogens. After stimulation with PAMPs, i.e. Poly I:C and PGN, primary HK macrophages express a large set of inflammatory genes including IL-1β, IL-6, IL-12 and TNF-α [17, 29, 31]. These cytokines can feed back on the macrophages themselves to regulate the expression of these genes and other effector molecules [17, 23, 27]. The strong induction of both trout IRF10a and IRF10b by the viral mimic Poly I:C at early (4 h) and late (24 h) time points, but only weak induction of IRF10b at 24 h post stimulation with bacterial PGN, suggests that IRF10 may have a particularly important role in antiviral defense. The weak induction of trout IRF10b by rIL-1β and rIL-6 (at 4 h only), and the lack of a response to stimulation with rTNF-α and two IL-12 isoforms indicates a limited role of the signaling pathways mediated by these proinflammatory cytokines on expression of the trout IRF10 paralogues. In contrast, IFN-γ up-regulates the expression of both trout IRF10 paralogues, suggesting an important involvement of type II IFN signaling on IRF10 induction. The role of type I IFN signaling on fish IRF10 expression remains to be determined.

The functional role of fish IRF10 as a transcriptional activator or repressor still remains to be established. Evidence in zebrafish revealed IRF10 may function as a negative regulator of the IFN response [13]. In salmonids, the presence of two isoforms of IRF10 may provide extra possibilities to fine tune the immune response. Trout IRF10a, with the potential to produce a truncated IRF10 without the DBD domain (discussed in Section 4.1), could function as a negative regulator of the full-length IRF10, i.e. the trout IRF10b, when both isoforms are expressed in the same cells. Thus, if one IRF10 isoform, i.e. IRF10b, functions as a repressor of the IFN response, the other, i.e. IRF10a, may function as an activator. Taking this into account, the different induction kinetics of IRF10a and IRF10b after Poly I:C stimulation is particular interesting (Fig 5). The predominant early induction of IRF10a (22.4-fold) over IRF10b (5.1-fold) at 4 h may favor IFN response, and the late strong induction of IRF10b at 24 h may inhibit IFN release to avoid an excessive immune response in rainbow trout.

### 4.4. IRF10 in antiviral defense in fish

In primary trout macrophage cultures, the (ds)RNA Poly I:C is a strong inducer of trout IFR10 expression compared to bacterial derived PGN. Poly I:C induced IRF10 expression is also seen in other fish species including swamp eel, Japanese flounder [12] and zebrafish [13]. Furthermore, IRF10 expression was strongly induced by viral infection, e.g. up to 368-fold by GCHV in grass carp HK, and by VHSV in rainbow trout and flounder kidney [12]. However, bacterial infections do have some small effects on IRF10 expression, e.g. up to 7.3-fold induction by *Edwardsiella tarda* and 4.2-fold by *Streptococcus iniae* infection in flounder kidney. This up-regulation is correlated with the induction of IFN-γ expression that is capable of increasing IRF10 expression, with type I IFN expression refractory to these two bacteria [12]. Viral infection induced IRF10 expression has also been seen in birds, where chicken IRF10 is strongly induced by infectious bursal disease virus [11]. Taken together these data suggest that IRF10 has an important role in host immunity, especially in immune defense to viral infection.

Interestingly, the expression of both trout IRF10 paralogues is induced by both pathogenic and non-pathogenic VHSV and IRF10b expression was induced earlier (at day 1) by the non-pathogenic VHSV at a time when there was no response in fish infected with pathogenic VHSV (Fig 7B). IFN-γ has been shown to be a strong inducer of IRF10 expression in trout primary macrophages. However, in the same VHSV infected samples, we observed a strong induction of IFN-γ expression by pathogenic virus, and at day 1 post infection IFN-γ expression is ~10 fold higher in fish exposed to the pathogenic VHSV compared to fish given nonpathogenic VHSV (un-published data). These data suggest that, at least in rainbow trout, IRF10 induction may represent an early host innate immune response to viral infection and this early response may be IFN-γ independent. The lack of an early induction of IRF10b by pathogenic VHSV could be part of an evasion mechanism [46].

In conclusion, we have cloned an IRF10 gene in grass carp and swamp eel and two genes in rainbow trout. The IRF10 genes have a general 8 exon/7 intron structure in fish and birds. The expression of IRF10 gene is highly induced by Poly I:C and viral infection, but is less responsive to PGN and pro-inflammatory cytokines, suggesting an important role in antiviral defense. Fish IRF10 gene expression may also be regulated at the post-transcriptional level by uORFs, with our functional studies confirming this for trout IRF10a *in vitro*. The two trout IRF10 paralogues cloned differ in a number of ways. They are differentially modulated, with trout IRF10b highly expressed in integumentary tissues compared to IRF10a, whilst IRF10a has the potential to make a truncated form.

## Supporting Information

S1 Fig Nucleotide and deduced amino acid sequences of grass carp *Ctenopharyngodon idella* IRF-10 cDNA (GenBank Acc. No. FJ556996). The nucleotides (upper row) and deduced amino acids (lower row) are numbered at the right side of sequences. The start and stop codons of the main ORF are in bold and boxed. An in frame stop codon upstream of the main ORF is boxed and shaded. Four potential upstream ORFs are in bold and underlined with their start and stop codons shaded. Three potential polyadenylation signals and an mRNA instability motif (ATTTA) are boxed and underlined, respectively.
(DOCX)

S2 Fig Nucleotide and deduced amino acid sequences of Asian swamp eel *Monopterus albus* IRF-10 cDNA (GenBank Acc. No. JX463268). The nucleotides (upper row) and deduced amino acids (lower row) are numbered at the right side of sequences. The start and stop codons of the main ORF are in bold and boxed. A potential upstream ORF is in bold and underlined with their start and stop codons shaded. The polyadenylation signal and mRNA instability motifs (ATTTA) are boxed and underlined, respectively.
(DOCX)

S3 Fig Comparison of the cDNA and deduced amino acid sequences of two IRF10 paralogues of rainbow trout. The cDNA sequences of trout IRF10a (A, Acc. No. HG917960) and IRF10b (B, Acc. No. HG917961) are aligned with their deduced amino acid sequences above or below the alignment, respectively. Dashes (-) indicate gaps introduced into the alignment. Identical nucleotide in trout IRF10b are represented with a vertical bar (|). The amino acids of IRF10b that differ from IRF10a are shaded. The nucleotide and deduced amino acids are numbered at the right of the sequences. The start and stop codons of the main ORF are in bold and boxed. An in frame stop codon upstream of each main ORF is boxed and shaded. Potential upstream ORFs are underlined with their start and stop codons shaded. The five Ws in the DNA binding domain are in bold. The binding sites of primers used for amplification of cDNA sequences are boxed.
(DOCX)

S4 Fig A Maximum Likelihood phylogenetic tree of vertebrate IRF members. The tree was constructed using an amino acid multiple alignment and the Maximum Likelihood method within the MEGA6 program (Tamura et al., 2013). The evolutionary history was inferred by using the method based on the JTT matrix-based model. The percentage of trees in which the associated taxa clustered together is shown next to the branches based on 5,000 bootstrap replications. The accession number for each sequence is given after the common species name and molecular type. The IRF10 molecules from trout, grass carp and swamp eel are in bold. A tentative grouping of vertebrate IRF subfamilies is shown on the right.
(PPTX)
