# Supplementary material for: Sequence and Expression Analysis of Interferon Regulatory Factor 10 (IRF10) in Three Diverse Teleost Fish Reveals Its Role in Antiviral Defense
Source: PLoS One. 2016 Jan 19;11(1):e0147181. doi: 10.1371/journal.pone.0147181 (PMC4718558; doi:10.1371/journal.pone.0147181)
Supplement: S1 Fig — The nucleotides (upper row) and deduced amino acids (lower row) are numbered at the right side of sequences. The start and stop codons of the main ORF are in bold and boxed. An in frame stop codon upstream of the main ORF is boxed and shaded. Four potential upstream ORFs are in bold and underlined with their start and stop codons shaded. Three potential polyadenylation signals and an mRNA instability motif (ATTTA) are boxed and underlined, respectively. (DOCX) [file pone.0147181.s001.docx]

TTCATCGAAAATTTCATTTTTTTTTTTA**ATGACCTTAGCACAAGTTTTATAG**AA**ATGTTGGTGTGTTACTGA**AGAACTGTTGTTTGGAAA 90

T**ATGAC**TGA**AGTTGTAGAGATAATTTGA**CGGTGTT**ATGTTTTAG**ATTG**ATG**GAAGACAGGTCGAGGCACATGCGACTGAGAGAATGGCTC 180

M E D R S R H M R L R E W L 14

ATAGCGCAGATAGACAGCGGCAAATACGCTGGACTAAGCTGGGAAAATGAGGAGAAAACCATGTTCAGAATTCCCTGGAAGCACGCCGCC 270

I A Q I D S G K Y A G L S W E N E E K T M F R I P W K H A A 44

AAACAGGACTACAGACAGAACCAGGACGCTGCGCTGTTTAAGGCATGGGCCATGTACAAGGGTAAATTTCAAGAGGGCAGGGACAAAGCA 360

K Q D Y R Q N Q D A A L F K A W A M Y K G K F Q E G R D K A 74

GATCCCTCTACTTGGAAAACACGCCTGCGTTGTGCACTCAACAAGAGCACAGACTTCCAGGAGGTTCCTGAGCGCAGTCAGCTGGATATC 450

D P S T W K T R L R C A L N K S T D F Q E V P E R S Q L D I 104

TCTGAGCCATACAAGGTCTACCGTATCCTGGACGACTCAGGAAGAGTGACAGAATATGCTGGAAACCCAGTGATATCACATGACAGCGAC 540

S E P Y K V Y R I L D D S G R V T E Y A G N P V I S H D S D 134

TGCAGCAAAGCCTTGCGAGAGACACGTCTGCCCATGCAGGAGGACAGTCCACTTGGTGACTCAAACAAAGGTGCTGGATGGAGTGTGAAT 630

C S K A L R E T R L P M Q E D S P L G D S N K G A G W S V N 164

GGCAGGTCGCATGCATGTCCCAGTACAGACACTAAAGCCTGCATTAACTCCAATCTTCAATCTGTTCCCATATATCCATCTCACGTCCCC 720

G R S H A C P S T D T K A C I N S N L Q S V P I Y P S H V P 194

ATATCTGACTGCCGTCTGGAGGTGCGTTTGTTCTATCATGGTAACCTGGTGCAAAGCCTCACAACTGCATCTCCAGACGGCTGCTTCATT 810

I S D C R L E V R L F Y H G N L V Q S L T T A S P D G C F I 224

CTGCAAGGCTGTGCTCCGGTAGGGAATGAGCGTATCTACGGGCCTTGTGAAGCCGAGAAGGTCTTCTTCCCCCGTCCAGACACCATCCGC 900

L Q G C A P V G N E R I Y G P C E A E K V F F P R P D T I R 254

CTGCCCCCGGGCATCGCTGAAGCCATGAGCCGTCTTCTGCCCCACCTGGAGAAAGGTGTGCTGGTGTGGGTGGCTCCAGATGGCGTGTTC 990

L P P G I A E A M S R L L P H L E K G V L V W V A P D G V F 284

ATCAAACGCTTCTGCCAGGGCCGCGTGTACTGGGACGGCCCATTGGCAGAGCACAGACAGAAACCAAACAAACTAGAGAGAGAGAGGACC 1080

I K R F C Q G R V Y W D G P L A E H R Q K P N K L E R E R T 314

TGTAAACTCCTGGATATGACCATCTTCATGCAAGAGTTACAAAGCCACCAGCAGGCCACTGGTCCCGAGCCACGATACACAGTGGACCTG 1170

C K L L D M T I F M Q E L Q S H Q Q A T G P E P R Y T V D L 344

TGCTTTGGAGAGGAGTTCCCTGACCCCAGCCAACCAAAGAACAAGAAGCTCATCACTGCACAAGTGATTCCTCTGTTTGCTGTAGAGTGT 1260

C F G E E F P D P S Q P K N K K L I T A Q V I P L F A V E C 374

CTCCGCAGGCATAATGCATCTAATAATGTGGAGATGAAGCAATCACCCCCACACAGGAAAACCAATGAT**TAG**ACACACCTTAATCAGAAC 1350

L R R H N A S N N V E M K Q S P P H R K T N D * 397

AGTACACATTTCAACATCCCTGTGTTTGCTTTCTCCAAATAATAATAATTAT**AATAAA**AATGACAATAAT**AATAAA**C**ATTTA**TTTTTCTG 1440

TAACTAACAGCAGTAG**AATAAA**ATATATGTTCACAAAAAAAAAAAAAAAAAAAAAAAAAAA 1501

**S1 Fig. Nucleotide and deduced amino acid sequences of grass carp *Ctenopharyngodon idella* IRF-10 cDNA (GenBank Acc. No. FJ556996).** The nucleotides (upper row) and deduced amino acids (lower row) are numbered at the right side of sequences. The start and stop codons of the main ORF are in bold and boxed. An in frame stop codon upstream of the main ORF is boxed and shaded. Four potential upstream ORFs are in bold and underlined with their start and stop codons shaded. Three potential polyadenylation signals and an mRNA instability motif (ATTTA) are boxed and underlined, respectively.
