# Supplementary material for: Sequence and Expression Analysis of Interferon Regulatory Factor 10 (IRF10) in Three Diverse Teleost Fish Reveals Its Role in Antiviral Defense
Source: PLoS One. 2016 Jan 19;11(1):e0147181. doi: 10.1371/journal.pone.0147181 (PMC4718558; doi:10.1371/journal.pone.0147181)
Supplement: S2 Fig — The nucleotides (upper row) and deduced amino acids (lower row) are numbered at the right side of sequences. The start and stop codons of the main ORF are in bold and boxed. A potential upstream ORF is in bold and underlined with their start and stop codons shaded. The polyadenylation signal and mRNA instability motifs (ATTTA) are boxed and underlined, respectively. (DOCX) [file pone.0147181.s002.docx]

CGGTCTGTCTGTGTCCGTGGTTCGAAGGTGAAGCTGTGGAGACCGAGAG**ATGTGTTGA**AG**ATG**GAAGAAGGAGCCAAGCTGCACCTGAAA 90

M E E G A K L H L K 10

GAGTGGCTGATCGGCCAGATAGAGAGCGGAAGGTATGAGGGACTGAGCTGGGAAAACGAGGAGAAAACCATGTTCAGGATCCCGTGGAAA 180

E W L I G Q I E S G R Y E G L S W E N E E K T M F R I P W K 40

CACGCAGCGAAGAAGGACTACAGGCAGACGGCGGACGCAGCTCTCTTCAAGGCCTGGGCTGTGTATAAAGGCAAGTACATTGAGGAAAGT 270

H A A K K D Y R Q T A D A A L F K A W A V Y K G K Y I E E S 70

GACCACCAAGAGCCAACCATGTGGAAAACCAGACTAAGGTGTGCCCTGAACAAGTCGACGGACTTCCAAGAGGTTCCCGAGCGCAACCAG 360

D H Q E P T M W K T R L R C A L N K S T D F Q E V P E R N Q 100

CTGGACATCACAGAACCCTATAAGGTCTATTGCATCCAGCAAGACAATCCACCAAAAACAAAACTGGAAATTAAGTCCTGTATCCTGAGC 450

L D I T E P Y K V Y C I Q Q D N P P K T K L E I K S C I L S 130

AAAGAGTCTCCCCAAGCAAATGATCAAGTGACTGTCCAGGGGTCTCCAGAGAAACCCAGCTTTCTGGACAGAGAGGTTCACTATCAAAAA 540

K E S P Q A N D Q V T V Q G S P E K P S F L D R E V H Y Q K 160

GAGTCATTTAAAGCAAAAGAAGAAGAAGAAAAACCAGTGTCAGGGGATCTGATGAGGGAGCACATGTACTGTGAGTTAAGAGATAAAAAG 630

E S F K A K E E E E K P V S G D L M R E H M Y C E L R D K K 190

CCTCAAAGTCAGGTCCCTAGTCCTATCACCTTCTTCAGCCCGCTTACTATATCAGACTTTCGTATGCAGGTGATGTTGTTGTATCAAGGC 720

P Q S Q V P S P I T F F S P L T I S D F R M Q V M L L Y Q G 220

CAGAAGGTAATGAAAGTGACCACCAAGAGCCCAGATGGGTGCTTCATTCTGCAAGGTCGTGTTCCCTTGGGAAATGAACGGATCTATGGG 810

Q K V M K V T T K S P D G C F I L Q G R V P L G N E R I Y G 250

CCCTGCAACGCCCAGCAGCTGTCGTTCCCTTCCCCGGCCTCTCTATCCTTGCCATCACACGTGGCTGAAACAATGGCTCGCCTTCTTTGT 900

P C N A Q Q L S F P S P A S L S L P S H V A E T M A R L L C 280

CACCTGGAGAGAGGTGTGCTTTTATGGGTGGCCCCAGAGGGGGTATTCATCAAGCGGTTCTGCCAGGGCAGGGTGTATTGGAGTGGTCCC 990

H L E R G V L L W V A P E G V F I K R F C Q G R V Y W S G P 310

ATGGCCCTACACACTGACCGGCCCAACAAACTGGAGCGAGAAAAGACCTTCAAACTGCTTGATATACCCACATTTCTCTCTGAGTTTCAG 1080

M A L H T D R P N K L E R E K T F K L L D I P T F L S E F Q 340

ACCTGTTTGCAGGGAAAGGGACCCTCACCTTCCTGTGAGATTGAGCTCTGCTTTGGAGAGGAGTATCCAGACCCCAGTGTACCAAAAACC 1170

T C L Q G K G P S P S C E I E L C F G E E Y P D P S V P K T 370

AGGAAGCTGATCATTGCACAGGTGGTGCCCTTGTTTGCAGTGGAACTGCTGCAGAAGTTCAACTTGGTAAAGACTGAGGAGAACCAGCTG 1260

R K L I I A Q V V P L F A V E L L Q K F N L V K T E E N Q L 400

ACTCTCAGCTCCAACACACAGAGAGAGAAGATG**TAG**GAATGCTGGGAAAAGGCTACACTGCACCAGAGCAGCCATGAACAATATACAAGT 1350

T L S S N T Q R E K M * 411

ATAGTACTGAACCTTAAGAGCGGTGTAAATACATTCTGTTGTCAGTGGAAATGTATATATATAATTGTAGACCTACTTGCTTTGCATAAC 1440

AGTAA**ATTTA**AAGCAATGTCCTTTAACTGTACCTTATACAGATTTTTTCTGTCCACTATGCAGCTAAACCTGCAAGAGTTAAAGCAAAGT 1530

AAAGCAAACATCTTGTTTAAACGAGCAAACAAATCATGTTAACATAGTATAAATGTAAAGTTTTTTCTGAATT**ATTTA**ATCTTAATCCCA 1620

TATTCAGCATATGTTTTACGTGACAGTTTAATTGTACAGTATTTTACATACTTTACATATCCAGAG**AATAAA**TTGACACCTTTAATTAAA 1710

TCTAAAAAAAAAAA 1724

**S2 Fig. Nucleotide and deduced amino acid sequences of Asian swamp eel *Monopterus albus* IRF-10 cDNA (GenBank Acc. No. JX463268).** The nucleotides (upper row) and deduced amino acids (lower row) are numbered at the right side of sequences. The start and stop codons of the main ORF are in bold and boxed. A potential upstream ORF is in bold and underlined with their start and stop codons shaded. The polyadenylation signal and mRNA instability motifs (ATTTA) are boxed and underlined, respectively.
