# Supplementary material for: Sequence and Expression Analysis of Interferon Regulatory Factor 10 (IRF10) in Three Diverse Teleost Fish Reveals Its Role in Antiviral Defense
Source: PLoS One. 2016 Jan 19;11(1):e0147181. doi: 10.1371/journal.pone.0147181 (PMC4718558; doi:10.1371/journal.pone.0147181)
Supplement: S3 Fig — The cDNA sequences of trout IRF10a (A, Acc. No. HG917960) and IRF10b (B, Acc. No. HG917961) are aligned with their deduced amino acid sequences above or below the alignment, respectively. Dashes (-) indicate gaps introduced into the alignment. Identical nucleotide in trout IRF10b are represented with a vertical bar (|). The amino acids of IRF10b that differ from IRF10a are shaded. The nucleotide and deduced amino acids are numbered at the right of the sequences. The start and stop codons of the main ORF are in bold and boxed. An in frame stop codon upstream of each main ORF is boxed and shaded. Potential upstream ORFs are underlined with their start and stop codons shaded. The five Ws in the DNA binding domain are in bold. The binding sites of primers used for amplification of cDNA sequences are boxed. (DOCX) [file pone.0147181.s003.docx]

A TGGTCATATTCGTTGTGCTGAAACATAGACATTTTATTTGATAACAATTTATTTTGATGAATCATTTTGTGTTAGCGTGTTATAGGTCTA 90

B -------------------------------------------------------||G|G||||---||||A|T|T|A|---||||C||T 29

A M V E L 4

A CTTGTGGTCTACACGTA--CAACTACAACTTT**TAG**AT-TTTTTGTTCGGAGCCTATTTTTTTCATATGTATTTTGAAG**ATG**GTGGAGCTG 177

B ||||||||||GT||**A|GGC|||TA|T|||||||GA**|CA|||||T||TTA||--||||CA|||A|A|C|||||||C|||**|||**|A|G||GCA 117

B M E K A 4

A V K K M R L R E **W** L I A Q I D S G K Y T G L T **W** E N E D K T 34

A GTCAAGAAAATGCGTTTGAGAGAGTGGCTGATAGCGCAGATAGACAGCGGAAAGTATACAGGACTTACCTGGGAGAACGAAGACAAAACT 267

B |G||A|||C||||A|C|||||||||||||||||||||||||||||||T||||||||CG|||||||C||||||||||||C|GA|||||||| 207

B G T N M H L R E **W** L I A Q I D S G K Y A G L T **W** E N Q N K T 34

A M F R I P **W** K H A A K Q D Y S L N E D A A L F K A **W** A V Y K 64

A ATGTTCAGGATCCCATGGAAACACGCGGCAAAACAGGACTATAGTCTGAACGAAGATGCAGCACTATTCAAGGCGTGGGCAGTGTATAAG 357

B |||||||||||||||||||||||||||||||||||||||||||A||||||T|||||||||||C||A|||||||||||||||||||||||| 297

B M F R I P **W** K H A A K Q D Y N L N E D A A L F K A **W** A V Y K 64

A G K Y R E G R D K A D P T T **W** K T R L R C A F N K S T D F K 94

A GGGAAGTATCGGGAGGGGAGGGACAAGGCAGATCCCACCACCTGGAAGACTCGTCTCCGCTGTGCCTTCAACAAGAGCACAGACTTCAAA 447

B |||||||||||A||||||||||||||||||||C|||||TT||||||||||||||A|||||||||||C||||||||||||||||||||C|G 387

B G K Y R E G R D K A D P T S **W** K T R I R C A L N K S T D F Q 94

A E V P E R S Q L D V S E P Y K V Y H I Q A E P E T G R D S E 124

A GAAGTCCCAGAGCGCAGCCAGTTAGATGTATCCGAGCCCTACAAGGTCTACCACATCCAGGCAGAGCCAGAGACAGGCAGAGACTCAGAA 537

B ||G||||||||||||||||||C|G||C||C||||||||||||||||C|||||GT||||||A|||||------|||||||||TG||||||| 471

B E V P E R S Q L D V S E P Y K A Y R I Q T A - - T R R C S E 122

A S P Q P E S Q M I I Q T S R S S V P L R N I V T H H P Q F G 154

A TCTCCTCAGCCTGAGAGTCAGATGATCATCCAGACCAGCCGCTCCAGTGTTCCACTGAGGAACATTGTCACACACCATCCACAGTTTGGC 627

B ||||||||AA||||A|||||||C|||A|||||||||||GA|||||||||C||AC||||||||||CCA||||||||||||||||||||||| 561

B S P E T E S Q A I I Q T R S S S A H L R N T I T H H P Q F G 152

A C N S E S E A R D G R V N S R E G L A G D H M Y Y W S N T G 184

A TGCAATAGTGAATCAGAGGCCAGGGATGGAAGAGTCAACTCCAGAGAGGGCTTGGCAGGGGACCATATGTACTACTGGTCCAACACAGGG 717

B |||C||||G||||||||||AA||||||||||||||||||T||||T|A|||T|||------||||||G|AC|||||||T|||||||||||| 645

B C H R E S E E R D G R V N P S G G L - - D H V H Y C S N T G 180

A N P Q R D G S A P L S I V V P T P Q I S D L R V R V C L F Y 214

A AATCCTCAGAGGGATGGCTCTGCTCCTCTCTCCATAGTCGTCCCTACACCACAGATCTCTGACTTGCGTGTGCGGGTGTGTCTGTTCTAT 807

B |C|||T||||T|||CA||||||||A|CT||||||||G||AG|||C||||||||||||||||||||C|||||||||||||||||||||||C 735

B T P Q M D S S A T F S I F S P T P Q I S D F R V R V C L F Y 210

A Q G Q L V V D V T T S T P D G C F L L Q G Q V P L G N E R I 244

A CAGGGCCAGCTAGTAGTGGATGTGACCACCAGCACCCCAGACGGCTGTTTCCTCCTGCAGGGTCAAGTGCCCCTGGGGAATGAGAGGATC 897

B ||||A||||||||||||||||||||||||||||||||||||T|||||||||A|T||A||T|||||G||||||||||||||C||||||||| 825

B Q D Q L V V D V T T S T P D G C F I L H G Q V P L G N E R I 240

A Y G P C T A Q Q V P F P P P G V I H L P P G I A E A M G R L 274

A TATGGACCCTGCACAGCCCAACAGGTCCCCTTCCCCCCCCCAGGGGTCATCCATCTTCCCCCGGGCATCGCTGAGGCCATGGGCCGCCTG 987

B |||||C|||||||||||||||||||||||||||||||||||||||||||||||C||G||||||||T||||||||||||||||A||||||| 915

B Y G P C T A Q Q V P F P P P G V I H L P P G I A E A M D R L 270

A L P H L E R G V L V W V A P D G V F I K R F C Q G R V Y W S 304

A CTGCCCCACCTGGAGAGGGGCGTCCTGGTGTGGGTTGCTCCAGACGGGGTGTTTATCAAGAGGTTCTGCCAGGGCAGGGTGTACTGGAGT 1077

B |||||||||T||||||||||T||||||||||||||G||||||||T||||||||||||||||||||||||||||||||||||||T|||||| 1005

B L P H L E R G V L V W V A P D G V F I K R F C Q G R V Y W S 300

A G P L A Q H T D R P N K L D R E R T C K L L D T A I F L K E 334

A GGCCCCCTGGCCCAACACACAGACAGGCCCAACAAACTGGACAGGGAGAGGACCTGCAAGCTGCTGGACACAGCTATATTTCTGAAGGAG 1167

B |||||T||||||||G||||||||||||||||||||G|||A||||A|||||||||||||||||||||A|TG||T||||||||||||||||| 1095

B G P L A Q H T D R P N K L N R E R T C K L L N A S I F L K E 330

A L Q D Y I Q G A G P K P R Y E I D L C F G E E F P D A S Q L 364

A CTCCAGGACTATATCCAGGGGGCGGGACCCAAACCTCGCTATGAGATTGACCTCTGCTTTGGTGAGGAGTTTCCTGACGCCAGCCAACTG 1257

B ||T|||||||T|C||A||||||||||||||||||||||||||||||||||||||||||||||||||||||||||C||||||||||CC||| 1185

B L Q D F L K G A G P K P R Y E I D L C F G E E F P D A S P L 360

A K T R K L I I A Q V V P L F A V N L L H R C L R M G T E G R 394

A AAAACCAGGAAACTGATCATTGCACAGGTGGTGCCCCTGTTTGCTGTCAACCTACTGCATAGGTGCCTGAGGATGGGGACAGAGGGGAGA 1347

B |||||||||||G|||||||||||||||||A|||||||||||C||||||||||||T||||G||||||||||||T||||||||**TAA**|A|||| 1275

B K T R K L I I A Q V V P L F A V N L L Q R C Q R L G P * 387

A P H L H T H K T M G E E G E G Q P H P L P Q R * 417

A CCGCACCTTCACACACACAAGACCATGGGGGAAGAGGGAGAGGGTCAGCCACACCCCCTGCCCCAGCGG**TAA**GGAGCCCAGGACACCCCT 1437

B ||A|G|||||||||||||||C|||||||||||G||||||A||||||||T||||A|||-|CT||||||AC||||||||||||||||||||| 1365

A CTGAGTAACCCCTGGGGAACATCACCAGTCCCCCCTGGTGGCCCCTCCTGTCCCAGCTTAGCCAGCCATGATGAGGGGTGAG 1519

B ||A|||||A|A||TA|||||||------|||||||||||A--|||||||||||T|||C|||||||||||||||||||||||| 1438

**S3 Fig. Comparison of the cDNA and deduced amino acid sequences of two IRF10 paralogues of rainbow trout.** The cDNA sequences of trout IRF10a (A, Acc. No. HG917960) and IRF10b (B, Acc. No. HG917961) are aligned with their deduced amino acid sequences above or below the alignment, respectively. Dashes (-) indicate gaps introduced into the alignment. Identical nucleotide in trout IRF10b are represented with a vertical bar (|). The amino acids of IRF10b that differ from IRF10a are shaded. The nucleotide and deduced amino acids are numbered at the right of the sequences. The start and stop codons of the main ORF are in bold and boxed. An in frame stop codon upstream of each main ORF is boxed and shaded. Potential upstream ORFs are underlined with their start and stop codons shaded. The five Ws in the DNA binding domain are in bold. The binding sites of primers used for amplification of cDNA sequences are boxed.
