# Supplementary material for: Sequence and Expression Analysis of Interferon Regulatory Factor 10 (IRF10) in Three Diverse Teleost Fish Reveals Its Role in Antiviral Defense
Source: PLoS One. 2016 Jan 19;11(1):e0147181. doi: 10.1371/journal.pone.0147181 (PMC4718558; doi:10.1371/journal.pone.0147181)
Supplement: S4 Fig — The tree was constructed using an amino acid multiple alignment and the Maximum Likelihood method within the MEGA6 program (Tamura et al., 2013). The evolutionary history was inferred by using the method based on the JTT matrix-based model. The percentage of trees in which the associated taxa clustered together is shown next to the branches based on 5,000 bootstrap replications. The accession number for each sequence is given after the common species name and molecular type. The IRF10 molecules from trout, grass carp and swamp eel are in bold. A tentative grouping of vertebrate IRF subfamilies is shown on the right. (PPTX) [file pone.0147181.s004.pptx]

## Slide 1
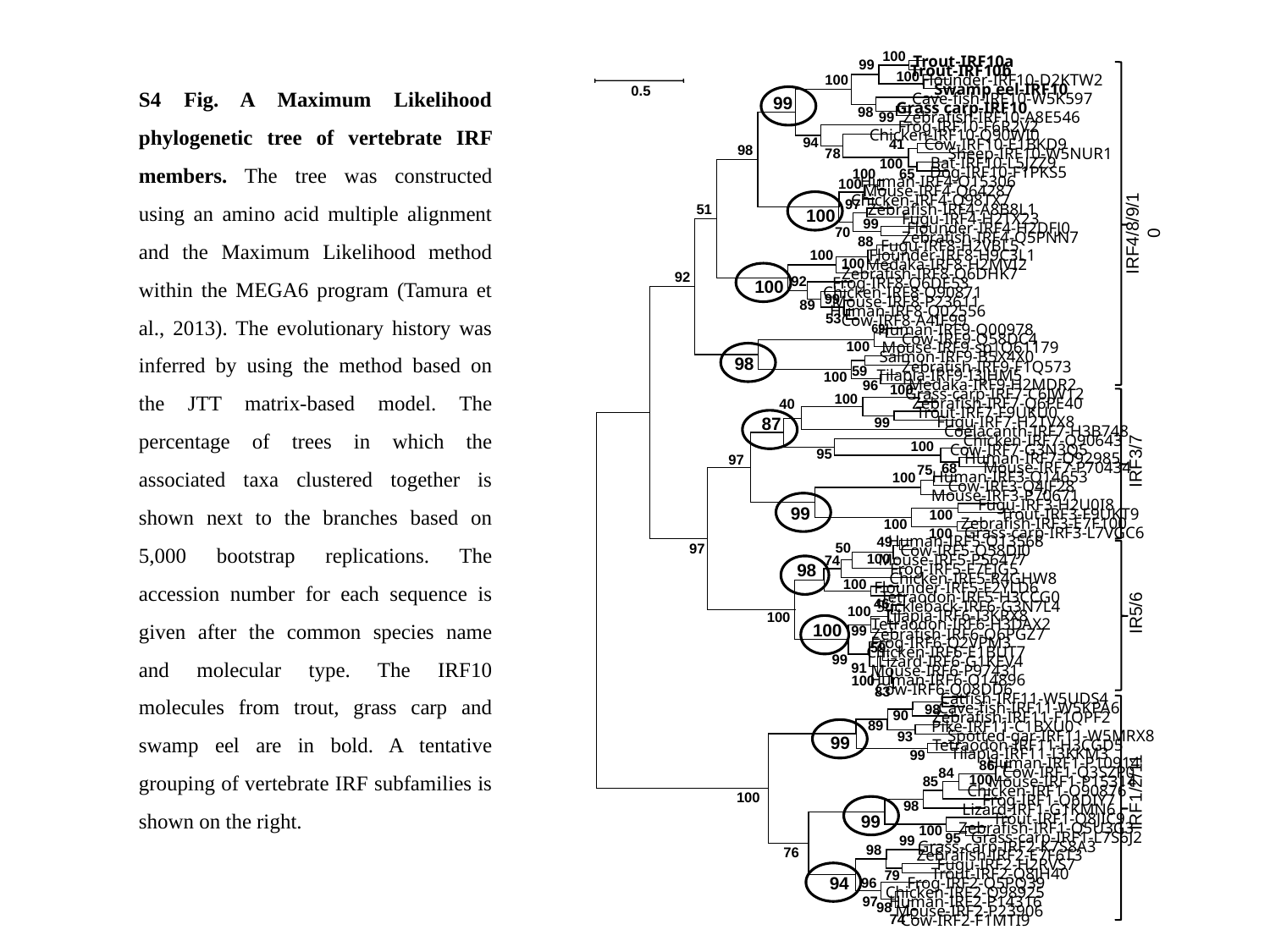

100
99
100
100
0.5
99
98
99
94
41
98
78
100
100
65
100
97
51
100
99
70
88
100
100
92
92
100
99
89
53
69
100
98
59
100
96
100
100
40
87
99
100
95
97
68
75
100
99
100
100
100
49
50
97
100
74
98
100
46
100
100
100
99
59
99
91
100
83
98
90
89
93
99
99
86
84
100
85
100
98
99
100
95
99
98
76
79
94
96
97
98
74
 Trout-IRF10a
 Trout-IRF10b
 Flounder-IRF10-D2KTW2
 Swamp eel-IRF10
 Cave-fish-IRF10-W5K597
 Grass carp-IRF10
 Zebrafish-IRF10-A8E546
 Frog-IRF10-F6R2V2
 Chicken-IRF10-Q90WI0
 Cow-IRF10-E1BKD9
 Sheep-IRF10-W5NUR1
 Bat-IRF10-L5JZZ9
 Dog-IRF10-F1PKS5
 Human-IRF4-Q15306
 Mouse-IRF4-Q64287
 Chicken-IRF4-Q98TX7
 Zebrafish-IRF4-A8B8L1
 Fugu-IRF4-H2TX23
 Flounder-IRF4-H2DFJ0
 Zebrafish-IRF4-Q5PNN7
 Fugu-IRF8-H2VBL5
 Flounder-IRF8-H9C3L1
 Medaka-IRF8-H2MVI2
 Zebrafish-IRF8-Q6DHK7
 Frog-IRF8-Q6DE53
 Chicken-IRF8-Q90871
 Mouse-IRF8-P23611
 Human-IRF8-Q02556
 Cow-IRF8-A4IF99
 Human-IRF9-Q00978
 Cow-IRF9-Q58DC4
 Mouse-IRF9-sp|Q61179
 Salmon-IRF9-B5X4X0
 Zebrafish-IRF9-F1Q573
 Tilapia-IRF9-I3JHM5
 Medaka-IRF9-H2MDR2
 Grass-carp-IRF7-C6JW12
 Zebrafish-IRF7-Q6PE40
 Trout-IRF7-F9UKU0
 Fugu-IRF7-H2TVX8
 Coelacanth-IRF7-H3B748
 Chicken-IRF7-Q90643
 Cow-IRF7-G3N3Q5
 Human-IRF7-Q92985
 Mouse-IRF7-P70434
 Human-IRF3-Q14653
 Cow-IRF3-Q4JF28
 Mouse-IRF3-P70671
 Fugu-IRF3-H2U0I8
 Trout-IRF3-F9UKT9
 Zebrafish-IRF3-E7F100
 Grass-carp-IRF3-L7VGC6
 Human-IRF5-Q13568
 Cow-IRF5-Q58DJ0
 Mouse-IRF5-P56477
 Frog-IRF5-F7EJG5
 Chicken-IRF5-R4GHW8
 Flounder-IRF5-F2YLD6
 Tetraodon-IRF5-H3CCG0
 Stickleback-IRF6-G3N7L4
 Tilapia-IRF6-I3KRX8
 Tetraodon-IRF6-H3DAX2
 Zebrafish-IRF6-Q6PGZ7
 Frog-IRF6-Q2VPM3
 Chicken-IRF6-E1BUT7
 Lizard-IRF6-G1KEV4
 Mouse-IRF6-P97431
 Human-IRF6-O14896
 Cow-IRF6-Q08DD6
 Catfish-IRF11-W5UDS4
 Cave-fish-IRF11-W5KPA6
 Zebrafish-IRF11-F1QPF2
 Pike-IRF11-C1BXU0
 Spotted-gar-IRF11-W5MRX8
 Tetraodon-IRF11-H3CGD5
 Tilapia-IRF11-I3KKM3
 Human-IRF1-P10914
 Cow-IRF1-Q3SZP0
 Mouse-IRF1-P15314
 Chicken-IRF1-Q90876
 Frog-IRF1-Q6DIY7
 Lizard-IRF1-G1KMN6
 Trout-IRF1-Q8JIC9
 Zebrafish-IRF1-Q5U3G3
 Grass-carp-IRF1-L7S6J2
 Grass-carp-IRF2-K7S8A3
 Zebrafish-IRF2-E7F6T3
 Fugu-IRF2-H2RVS7
 Trout-IRF2-Q8JH40
 Frog-IRF2-Q5PQ39
 Chicken-IRF2-Q98925
 Human-IRF2-P14316
 Mouse-IRF2-P23906
 Cow-IRF2-F1MTI9
IRF4/8/9/10
S4 Fig. A Maximum Likelihood phylogenetic tree of vertebrate IRF members. The tree was constructed using an amino acid multiple alignment and the Maximum Likelihood method within the MEGA6 program (Tamura et al., 2013). The evolutionary history was inferred by using the method based on the JTT matrix-based model. The percentage of trees in which the associated taxa clustered together is shown next to the branches based on 5,000 bootstrap replications. The accession number for each sequence is given after the common species name and molecular type. The IRF10 molecules from trout, grass carp and swamp eel are in bold. A tentative grouping of vertebrate IRF subfamilies is shown on the right.
IRF3/7
IR5/6
IRF1/2/11
